# Addressing bioreactor hiPSC aggregate stability, maintenance and scaleup challenges using a design of experiment approach

**DOI:** 10.1186/s13287-024-03802-4

**Published:** 2024-07-02

**Authors:** Haneen Yehya, Sofija Raudins, Roshan Padmanabhan, Jan Jensen, Michael A. Bukys

**Affiliations:** 1 Trailhead Biosystems, 23215 Commerce Park, Beachwood, OH 44122 USA; 2 Cleveland State University, 2121 Euclid Ave, Cleveland, OH 44115 USA

**Keywords:** iPSC, Human induced pluripotent stem cell, Pluripotent state, Bioreactor, DoE, Culture media, Suspension culture, Aggregates, Aggregate size, Aggregate stability, Proliferation

## Abstract

**Background:**

Stem cell-derived therapies hold the potential for treatment of regenerative clinical indications. Static culture has a limited ability to scale up thus restricting its use. Suspension culturing can be used to produce target cells in large quantities, but also presents challenges related to stress and aggregation stability.

**Methods:**

Utilizing a design of experiments (DoE) approach in vertical wheel bioreactors, we evaluated media additives that have versatile properties. The additives evaluated are Heparin sodium salt (HS), polyethylene glycol (PEG), poly (vinyl alcohol) (PVA), Pluronic F68 and dextran sulfate (DS). Multiple response variables were chosen to assess cell growth, pluripotency maintenance and aggregate stability in response to the additive inputs, and mathematical models were generated and tuned for maximal predictive power.

**Results:**

Expansion of iPSCs using 100 ml vertical wheel bioreactor assay for 4 days on 19 different media combinations resulted in models that can optimize pluripotency, stability, and expansion. The expansion optimization resulted in the combination of PA, PVA and PEG with E8. This mixture resulted in an expansion doubling time that was 40% shorter than that of E8 alone. Pluripotency optimizer highlighted the importance of adding 1% PEG to the E8 medium. Aggregate stability optimization that minimizes aggregate fusion in 3D culture indicated that the interaction of both Heparin and PEG can limit aggregation as well as increase the maintenance capacity and expansion of hiPSCs, suggesting that controlling fusion is a critical parameter for expansion and maintenance. Validation of optimized solution on two cell lines in bioreactors with decreased speed of 40 RPM, showed consistency and prolonged control over aggregates that have high frequency of pluripotency markers of OCT4 and SOX2 (> 90%). A doubling time of around 1–1.4 days was maintained after passaging as clumps in the optimized medium. Controlling aggregate fusion allowed for a decrease in bioreactor speed and therefore shear stress exerted on the cells in a large-scale expansion.

**Conclusion:**

This study resulted in a control of aggregate size within suspension cultures, while informing about concomitant state control of the iPSC state. Wider application of this approach can address media optimization complexity and bioreactor scale-up challenges.

**Supplementary Information:**

The online version contains supplementary material available at 10.1186/s13287-024-03802-4.

## Background

Human pluripotent stem cells (hPSCs) can proliferate continuously and have the capacity to differentiate into any human cell type. Upon differentiation, this unlocks their potential to treat various clinical indications such as diabetes, orthopedic injuries, neurological, cardiovascular diseases, and others by supplying cells needed to restore tissue function. Induced pluripotent stem cells (iPSCs) or reprogrammed cells generated from mature specialized cells [1], are becoming the focus in the field of regenerative medicine, as they are not restricted by ethical concerns of embryonic stem cells but display a similar differentiation and growth capacity as previously used embryonic stem (ES) cells. While no current FDA-approved iPSC cell therapy exists, many are in clinical trials. Therapeutic applications of iPSCs in regenerative medicine typically depend on the availability of 10^8^–10^10^ clinical grade cells per patient, where such material is manufactured using a current Good Manufacturing Practice (cGMP) process and performed within an isolated production environment to assure patient safety [2–4].

Conventional adherent culture flasks are impractical for large scale production as they require frequent manual intervention. Adherent cultures have a limited ability to scale up and result in high batch-to-batch variability and a lack of cost effectiveness. Alternatively, continuously stirred-tank suspension bioreactors provide an hiPSC 3D culture that generates aggregates that are more biologically comparable to an in vivo environment [5]. In addition, bioreactors can provide a continuous monitoring of environmental factors including temperature, pH, oxygen, and nutrients in a sterile environment required to produce clinical grade cells. Maintaining pluripotent state in large-scale suspension environment will improve reproducibility and cell quality, but it requires control over media composition, aggregation and physicochemical stresses exerted on cells.

iPSC expansion in bioreactors presents several challenges including cell clumping, shear stress, complex media composition and cost. Cell clumping or aggregate fusion can cause heterogenous cell populations to arise [6]. Shear stress leads to aggregate breakage, DNA-breaks, resulting in cell death and karyotypic instability [7]. An environment that can prevent unwanted cell adhesion and maintain aggregate stability while maintaining pluripotency for manufacturing expansion is in demand. Cell State control is particularly relevant, as heterogeneous populations presents a serious risk, as incomplete differentiation impacts both clinical safety and potency [6]. While simple elements such as temperature and pH can be controlled and monitored, molecular component interactions, concentration sensitivity, chemical stability of additives, when combined with physical stresses, mechanical forces present all combine into a very challenging production problem; this also presents an opportunity to identify which key critical process parameters truly govern culture performance [8]. For instance, although recent studies confirm that mechanical stimuli affect iPSCs during their differentiation [9, 10], mechanosensing signaling is not well understood especially in terms of stem cell mechanobiological interactions within the physical environment, of a bioreactor [11, 12].

A critical challenge to generating well-controlled studies assessing higher-order interactions is characterizing the complex mixture of variable that affect the molecular and functional response of iPSCs [12]. Over the last 18 years, exhaustive optimization has been done by multiple groups mainly using various adherent hiPSC culture media. This has led to the identification of FGF2 [13] and TGFb1 as the two critical growth factors in the Essential 8 (E8) medium formulation. E8 is a further modification identifying the essential components from the original TeSR medium which pioneered early pluripotency studies. Through an intermediary B8 medium which is a cost reduced optimization from the E8 medium [14]. Currently, there is broad focus on determining defined, stable, cost-effective solutions for iPSC culture maintenance and growth. One of the main improvements in the E8 formulation is the absence of bovine serum albumin (BSA) component, since it is a xeno component that has issues with consistencies. Pairwise dropout testing done in the E8 optimization effort uncovered that β-mercaptoethanol (BME) without BSA caused toxicity, but with the absence of BME, BSA was able to be eliminated from the medium. This finding highlights the complexity of doing optimization experiments without studying the interactions and non-linear effects. A key factor in obtaining well-founded results is understanding interaction effects and reducing the variability of environmental factors in the process [14]. Limited research evaluated the media needs for suspension culture that can address the shear stress generated from a bioreactor and the absence of substrate attachment in a 3D environment.

Here, we demonstrate a systematic approach maintaining iPSC aggregates in a uniform controlled stable size within a bioreactor. Heparin sodium salt (HS), polyethylene glycol (PEG), poly (vinyl alcohol) (PVA), Pluronic F68 (Pluronic F68) and dextran sulfate (DS) are compounds commonly used in biomedical and pharmaceutical applications. They have versatile properties such as reducing shear stress by decreasing surface tension of the media, enhancing extracellular matrix and cell membrane interaction, increasing aggregate stability, and preventing aggregate fusion [15–17]. Using a design of experiment (DoE) factorial study we develop process understanding of the complex interactions between these components. The DoE approach identifies statistically significant interactions resulting in a media formulation that is reproduceable by limiting variation bias. Models generated using these 5 factors can be optimized for three major cell culture criteria: (1) growth rate or doubling time, (2) pluripotency maintenance, and (3) aggregate stability. These variables are shown to be independent, and optimization for each attribute is related to different culture conditions. Consequently, attaining ideal culture conditions across the desirability criteria spectrum is an exercise based on compromise.

## Methods

### Generating DoE designs and perturbation matrixes

The DoE runs were computer generated from MODDE software (Sartorius Stedim Data Analytical Solutions, SSDAS) using D-optimal interaction designs. All components tested and outputs measured were manually inputted into Design Wizard in MODDE. Factors known to impact the aggregate stability in suspension culture were chosen for the design. Reagent concentration ranges were based on previous publications [15–17]. Design runs were chosen to have 16 reactions conditions with the addition of 3 center point conditions. One of the reactions had no additives, which is the E8 bioreactor. Examples of reaction additives of different bioreactors are shown in (Additional file 1: Fig. S1A, B). The highest G-efficiency design was chosen from the DoE designs generated by the software. Media reactions were made manually and filtered before use. Negative log transform was only used to normalize distribution for flow data sets for OCT4 and TRA-1-60 to meet normality assumptions. Assay and media preparation along with detailed methods are described in the supplemental information. qPCR primers, antibodies and reagents used are listed in (Additional file 1: Tables S1 and S2).

### Human induced pluripotent stem cell culture

Cells were maintained at 37 °C and 5% CO_2_. E8 medium was purchased ready in solution. Other medium components were purchased as powder. Prior to the assay, cells were grown to 60–70% confluence after 4 days of culture on vitronectin coated 6 well plates. Cells were dissociated with TrypLE, for 3 min at 37 °C and resuspended in E8 medium, transferred to 50 ml conical tubes, and centrifuged at 400 × g for 6 min. The pellet was resuspended in E8 and 10 µM Y-27632 ROCK inhibitor. 11 million cells were seeded in a 100 ml bioreactor with the corresponding media.

### Cell counting and aggregate size

Samples from the PBS vertical wheel bioreactors (PBS Biotech FA-0.1-D-001) were taken after mixing using a pipette to avoid sampling bias and gradient formation after settling. 3 ml total was sampled daily for 3 samples of 1 ml for cell count. The cells were first dissociated using Accutase and incubated for 10 min. Cells were then quenched with E8 then centrifuged at 400 × g for 6 min. The pellet was resuspended in the same volume of PBS. Samples were then analyzed for total cell count using Attune flow. Triplicate counts were collected for each bioreactor for each day of culture. Duplicate samples were collected for aggregate imaging of 500 µl and put on a 24 well plate. EVOS 7000 was used for bright field images of the aggregates. ImageJ was then used to analyze aggregate size and distribution. A minimum of 30 aggregates were measured for each bioreactor every day of the culture. This data were then analyzed for standard deviation on excel and growth rate using GraphPad Prism9. The cell counts were also analyzed for doubling time using GraphPad Prism9. Average data from aggregate size and counts were then inputted into the response tab in MODDE. Using the aggregate size generated from day 1 from all bioreactors and their corresponding cell growth rate, a predicted aggregate size was calculated for day 3 and compared to the actual size measured. The predicted aggregate size was calculated from theoretical aggregate volume. Assumptions made were the size of the cell diameter being 10 µm based on in house measurements and 64% random aggregate packing density based on empirical observations and simulation experiments [18].

Aggregate size measured on Day 1 was used to calculate aggregate volume using equation:

1 $${\text{V}}_{{1}} = \, \left( {{4}/{3}} \right) \cdot \, \pi \cdot {\text{R}}^{{{3} }}$$

The aggregate volume was divided by the individual cell volume defined as

2 $${\text{V}}_{{\text{c}}} = \, \left( {{4}/{3}} \right) \cdot \pi \cdot \left( {{1}0/{2}} \right)^{{3}} \, = {523}.{6}$$

The division was multiplied by the packing density to obtain cell number in an aggregate volume:

3 $${\text{N}}_{{1}} = {\text{V1}}/{\text{V}}_{{{\text{c}} }} \cdot 0.{64}$$

The predicted cell number of an aggregate after 3 days in culture was calculated from the cell growth rate measured from daily count (K) using the equation below:

4 $${\text{N}}_{{{\text{3P}}}} = {\text{ N}}_{{1}} *{\text{exp}}\left( {{\text{K}}*{3}} \right)$$

From the cell number, the predicted aggregate volume (V_3P_) was calculated again from the first equation. We defined the difference between the predicted and actual size as “Aggregate % Error”. This is calculated by subtracting the predicted from the actual average size measured and divided by the actual value:

5 $${\text{Aggregate }}\% {\text{ Error }} = \, \left( {{\text{V}}_{{3}} - {\text{V}}_{{{\text{3P}}}} } \right)/{\text{V}}_{{3}}$$

### Flow cytometry and qPCR testing

A 3 ml sample was taken from each bioreactor on day 4 of the culture for qPCR testing. RNA samples were dissolved in Trizol and extracted according to manufacturer’s protocol. Quantification of RNA was performed on epoch reader. A high-Capacity cDNA RT Kit was used for reverse transcription of RNA samples of each bioreactor. Triplicate samples of cDNA were obtained from triplicate samples of each bioreactor. Data collection was performed using QuantStudio for qPCR testing. The runs were performed per the manufacturer’s protocol and recommendations. The primers used were NANOG, SOX2, OCT4 and GAPDH (Additional file 1: Table S1). The data set obtained was then exported to Excel and normalized against corresponding housekeeping gene GAPDH. Final expression levels were expressed as 1/(2^DCrt^) × 1000 and inputted into the MODDE software. A 3 ml sample was taken from each bioreactor on day 4 of the culture for flow testing. The aggregates were dissociated with Accutase for 10 min into single cells. Cells were resuspended in PBS and divided into samples for intracellular staining and cells for extracellular staining. Sample for intracellular staining were fixed with a live/dead stain FVS 780**,** then permeabilized and stained for OCT4 and SOX2 using Attune™ Flow Cytometry (Additional file 1: Table S2).

### Immunofluorescent staining

hiPSC aggregates from day 4 were dissociated with Accutase and plated onto a Vitronectin treated 24 well plate and grown for 1 day. Cells were fixed with 5% PFA in DPBS for 15 min at room temperature, permeabilized and blocked with a blocking buffer solution (1% BSA and 0.1% Tween-20) for 60 min at room temperature and stained with primary antibodies at 1:500 (OCT4, SSEA4, SOX2, NANOG) (Additional file 1: Table S2) overnight. Cells were then washed with DPBS and stained with secondary antibodies (488-Donkey anti- Mouse and 594-Donkey anti-Rabbit) for 60 min. Cells were washed three times with DPBS (5 min each) and stained with DAPI. Plates were imagined using EVOS M7000 Imaging System microscope.

### Tri-lineage differentiation

iPSCs that were maintained and cultured in the optimized medium were cryopreserved in 10% DMSO and recovered on 6-well plates to be tested for differentiation potential. The cells were differentiated using the STEMdiff™ Trilineage Differentiation Kit protocol (STEMCELL Technologies). Immunofluorescence was carried out using antibodies for endoderm, ectoderm, and mesoderm lineage specific markers.

### Karyotyping analysis

Karyotyping was characterized by WiCell Research Institute on live cells that were maintained in optimized medium. The results showed normal karyotype with no clonal abnormalities detected at the stated band level of resolution (425–450).

### RNA isolation, library preparation and RNA-seq

The RNA was qualified by using Qubit™ RNA BR Assay Kit (ThermoFisher- Catalogue No- Q10211). Total RNA was isolated using MagMAX™-96 Total RNA Isolation Kit (ThermoFisher Scientific) according to the manufacturer’s instructions. RNA quality was validated using 4200 TapeStation System (Agilent Technologies). Enrichment of polyadenylated RNA and library preparation were performed using Illumina Stranded mRNA Prep (illumina) using the reagents provided in an Illumina® TruSeq® Stranded mRNA library prep workflow. The library underwent a final cleanup using the Agencourt AMPure XP system (Beckman Coulter) after which the libraries’ quality was assessed using a 4200 TapeStation System (Agilent Technologies).

For all samples, the sequencing was done at Genewiz from Azenta Life Sciences. The quality trimming and alignment of the samples were conducted using the nextflow nf-core/rnaseq pipeline (version 3.6). The pipeline incorporated Trim Galore (v.0.6.7) for adaptor trimming and quality control. The trimmed RNAseq reads were then mapped to the Homo sapiens GRCh38 genome annotation utilizing STAR (v 2.6.1). Datasets underwent filtration to eliminate low counts (< 10 reads), and differentially expressed genes were identified using the “DESeq2” (v 1.40.2) package in “R” (4.3.1). The heatmaps was created using the package “pheatmap” (v1.0.12).

### Statistical analysis

Data were analyzed and graphed in Excel, R, GraphPad Prism9 and MODDE software. Modeling of design space was performed using MODDE software. Three replicated center point experiments with all five additives at mid-concentrations were used and split between the batches for normalization and model evaluation purposes. The model responses for each parameter were statistically evaluated for model reproducibility, model validity, fit (R2) and prediction precision (Q2). Comparisons were conducted via ANOVA test with a significant difference defined as *P* < 0.05. The number of bioreactor reactions was predetermined by the MODDE generator. The experiments were not randomized for blocks before testing.

## Results

### Using a DoE approach to optimize pluripotent aggregate expansion and cell state

**Fig. 1:**
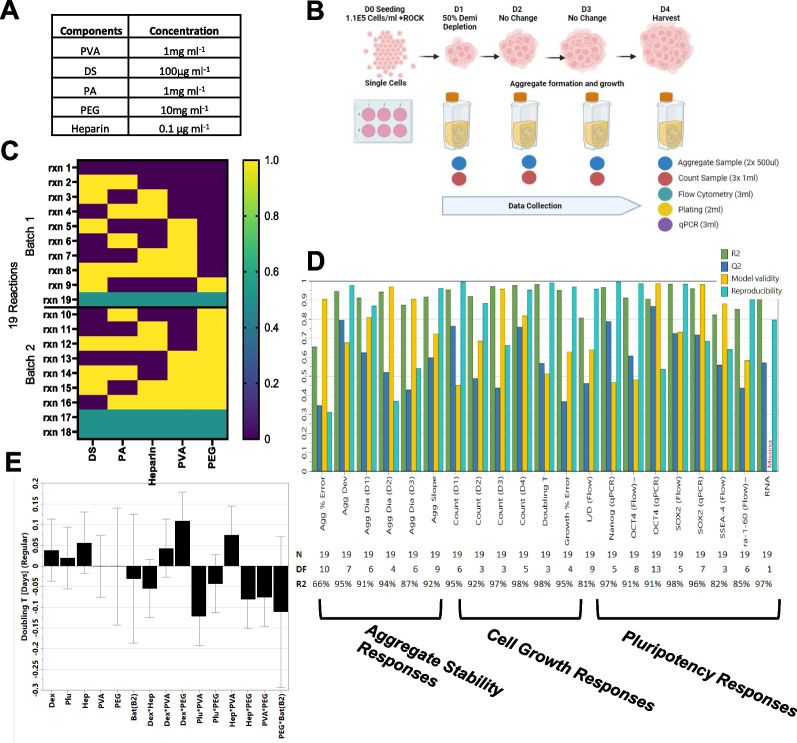
Bioreactor-based optimization of human pluripotent stem cell culture medium additives. Results are normalized to 3 replicate center point experiments that have all components at mid-concentration levels. Reactions are completed using NCRM-1 hiPSC line with a 4-day aggregate growth experiment. **A** Concentrations of medium formula additives used in experiment. **B** Bioreactor assay schematic shows what data are collected throughout the experiment. Initial seeding density was 1.1E5 cells/ml. Figure created with BioRender.com. **C** Design of experiment in a factorial design of 5 media additives. **D** The DoE model parameters: model validity, reproducibility, fit (R2), and prediction precision (Q2). **E** Coefficient plot of doubling time based on the response results of all variables and interactions detected. These bar coefficient plots provides graphical presentation of the significance of the model terms

D-optimal DoE interaction designs were used to compress the number of experimental runs compared with a full factorial design. Design compression allows determination of interactions and predictions within the design space explored within the concentration range used (i.e., ‘‘known space’) [19]. Multiple high-molecular weight polymers have previously been shown to impact pluripotent stem cell cultures, but combinatorial assessments have not been performed to any large extent. Here, we investigated dextran sulfate (DS), Heparin sodium salt (HS), poly (vinyl alcohol) (PVA), Pluronic acid F68 (PA) and polyethylene glycol (PEG) (Fig. 1A). These factors and their concentration range were chosen based on previous publications about their impact on the iPSC aggregates growth and maintenance. Previous work showed that expansion of hPSCs in stirred-type bioreactors was improved by the addition of Pluronic F68 acting as non-ionic shear protectant [15]. PA has an average molecular weight of 8400 Da and is a triblock copolymer of poly (ethylene oxide)–poly (propylene oxide)–poly (ethylene oxide). PA was also demonstrated to restore cell growth and viability in cell cultures [20]. PVA is a biocompatible synthetic polymer with a molecular weight that ranges between 31,000 and 50,000. It is commonly used in the pharmaceutical and food industries [21]. In a recent study, researchers replaced serum albumin with PVA to develop a culture system that supports long-term expansion of functional mouse hematopoietic stem cells (HSCs) [22]. PVA was also evaluated in hiPSC culture to promote proliferation. At 1 mg/mL PVA resulted in maximal cell density within the range tested from 0.1 to 10 mg/ml [23]. PEG is a polymer based on the -CH2CH20- repeat unit that comes in different size ranges [24]. Similarly, to PVA, PEG is approved for drug and pharmaceutical applications. It functions as an aggregation inhibitor/emulsifying agent that may improve protein stability [16]. DS is a polysulphate that may increase aggregate stability and cell growth. Heparin sodium salt supports hiPSC growth due to FGF2 stabilization. FGF2 degrades relatively quickly at 37 °C [17, 25]. Dextran sulphate has been commonly used in the biopharmaceutical industry to prevent cell aggregation. It is a poly-sulphated compound and was recently reported to control aggregate size and shape properties of hiPSCs, without compromising the maintenance of pluripotency [5, 26].

We generated D-optimal DoE interaction design to evaluate these effectors. Cultures were maintained for four days in a suspension environment of a 100 ml vertical wheel bioreactor (Fig. 1B). Reactors were initially seeded from 60 to 70% confluent hiPSCs pre-cultured on coated vitronectin plates [24] that were dissociated into single cells with TrypLE and seeded at 1.1E5 cells/ml in the presence of Rho kinase inhibitor Y-27632 for the first 24 h. Daily aggregate samples were collected to obtain images. On the final day of culture, RNA was extracted and used in cDNA synthesis. Cell samples for flow, counts and plating were digested using Accutase and processed for FLOW analysis using cell state specific antibodies.

Pluripotency data were assessed at the end of the experiment through Immunohistochemistry (IHC), flow cytometry and qPCR testing. The experiment was divided into two batches of experiments to be run at different times (Fig. 1C). Three replicated center point experiments with all five additives at mid-concentrations were used and split between the batches for normalization and model evaluation purposes. The optimization criteria were based on multiple attributes related to aggregate stability, cell growth and pluripotency (Fig. 1D). Mathematical models were generated and tuned for maximal predictive power and fit. The model responses for each parameter were statistically evaluated for model reproducibility, model validity, fit and prediction precision. R2 is the model fit, Q2 is the estimate of future prediction, the model validity is based on statistically significant model problems and outliers, and reproducibility is based on replicates where greater than 0.5 values indicate acceptable reproducibility of results. All response variables measured had an R2 value above 0.5 indicating a model with high significance. The model had model validity above 0.25 indicating the absence of problems related to outliers and transformation issues (Fig. 1D).

Inspection of the response models reveal insights on the degree of single/combinatorial control of key culture parameters. Invariably, coefficient listings of responses revealed that most significant predictors of culture behavior are contributed by factor interactions (Fig. 1E).

All bioreactor runs were performed using E8 basal medium for pluripotency maintenance creating a suitable bioreactor growth assay in which the impact of additives upon iPSC cell aggregate stability and growth could be measured daily (Additional file 1: Fig. S2A). The assay conditions were based on our previous work evaluating process parameters such as bioreactor seeding density, digestion frequency (Additional file 1: Fig. S2B), single cell dissociation (Additional file 1: Fig. S2C), passage time (Additional file 1: Fig. S2D), plate coating, and bioreactor speed. Using the data obtained from the short-term growth assay (Additional file 1: Fig. S2E), prediction models were constructed using computer modeling software. Response variables included aggregate diameter sizes, cell concentration, growth rates, pluripotency marker expression, cell viability and aggregate variability. Each response model estimates main factor effects along with interactions between factors.

### Impact of additives on suspension growth kinetics

**Fig. 2:**
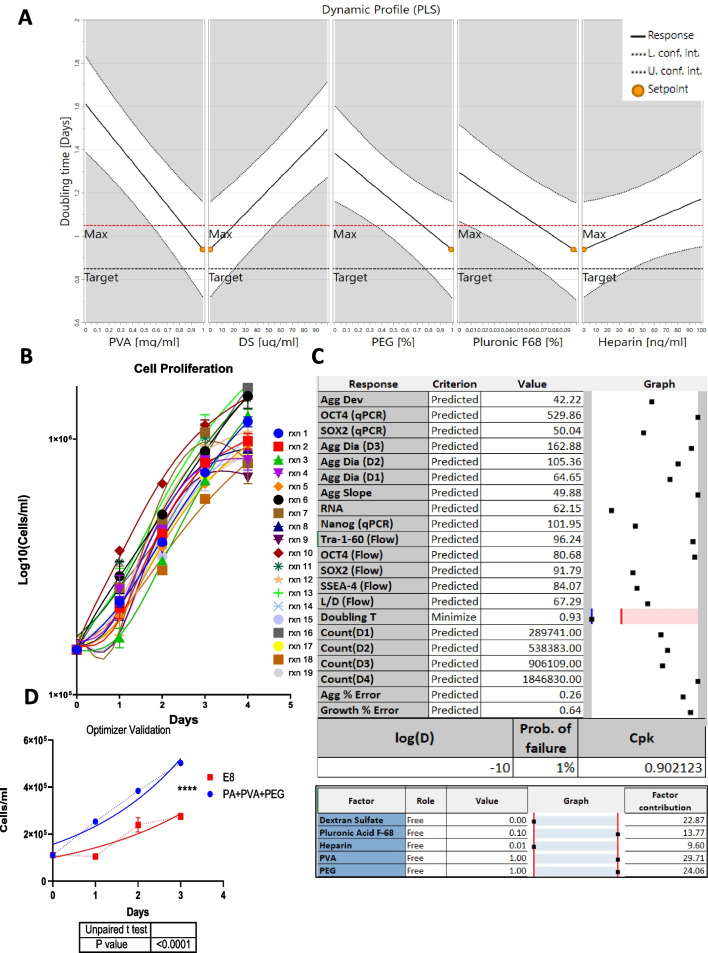
Modeling optimal proliferation within a bioreactor. **A** Dynamic Profile plot (PLS) for optimizer setpoint. **B** Measured growth curves throughout 4-day bioreactor run. **C** Optimizer results of doubling time parameter. **D** Validation of the doubling time optimizer as compared to E8. All charts show individual points with mean ± SD. *P < 0.05, **P < 0.01, ***P < 0.001, ****P < 0.0001

Various cell culture additives have been used to protect cells from environments that involve agitation-aeration cell damage [15, 20, 27–29]. The additives selected in this design are components known to have a positive impact on growth and protection from shear stress. An optimizer setpoint dynamic profile was performed to detect main factor contributions (Fig. 2A) using doubling time data collected over 4 days of culture in bioreactors (Fig. 2B). Desirability criteria were set for the minimization of doubling time (i.e. maximal growth rate, Fig. 2C). Optimizer results detected read out conditions revealed that Pluronic F68, PVA and PEG all contribute to increased proliferation. The process capability index (Cpk) obtained from this optimizer was 0.9 (Fig. 2C) with a probability of failure of 1%. According to six sigma statistics, a process is said to be “capable” if its Cpk is close to 1, meaning the target is centered between the specified limit under the natural tolerance 6σ [30]. Modeling terms for maximal growth rate control include interaction terms between the additives.

While interpreting and confirming a triple interaction can be complex, the obtained results from the model optimizer and coefficients plot confirm a combined effect of these three additives that is not simply additive through independent factor contributions (Fig. 1E) but rather there is a synergistic relationship observed contributing to the desired overall growth in this suspension system.

hiPSC culture without daily media changes presented caveats related to impact of increased cell growth eventually plateauing due to nutrients limitation. Also, production of glycolytic waste products such as lactate will reduce the medium pH, negatively impacting pluripotency, and continuous growth. For this purpose, the original seeding density of cells was at 1.1E5 cells/ml and the assay was revalidated at day 3 in addition to day 4 for many variables measured. Validation experiments compared the optimized medium to the control E8 medium without additives (Fig. 2D). The difference between the growth rates of the two media confirms the advantage of the additives (k = 0.4) over the control (k = 0.35).

### Optimization of pluripotency maintenance

**Fig. 3:**
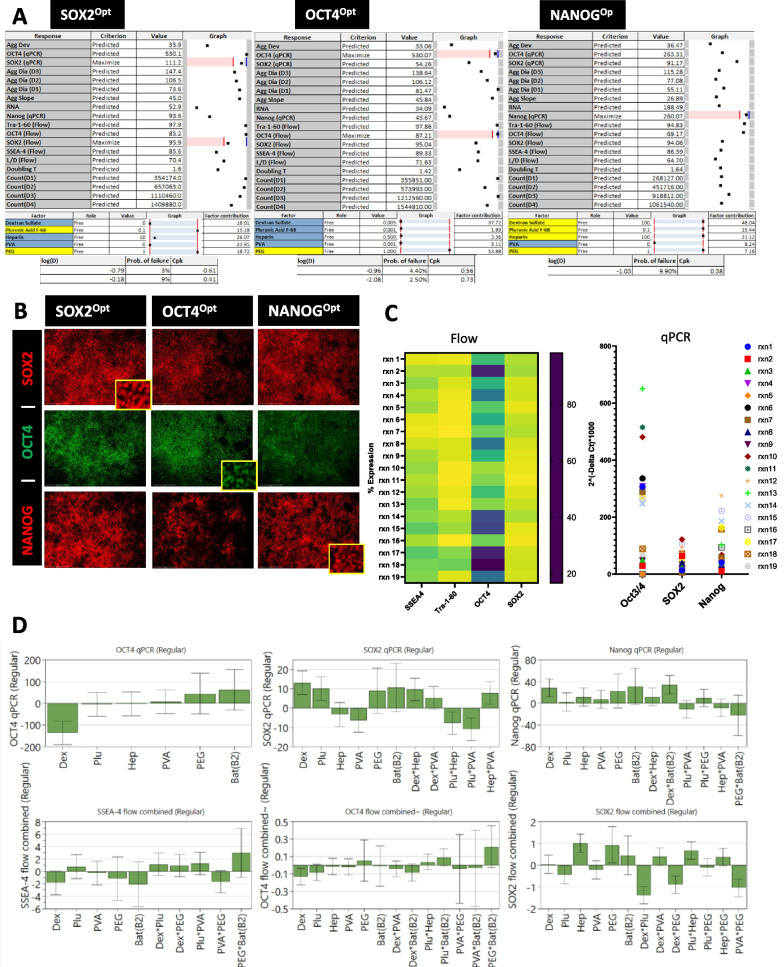
Modeling pluripotency maintenance within bioreactor. **A** MODDE model optimizers for pluripotency markers SOX2, OCT4 and NANOG. **B** Immunocytochemistry analysis of iPSC within the corresponding predicted optimizer conditions. **C** Variability measured between bioreactor conditions as determined using flow (heat map) and qPCR (graph). **D** Coefficients plots showing regulation of the pluripotency markers measured. These bar coefficient plots provide graphical presentation of the significance of the model terms

Attaining maximal growth conditions of iPSCs is not meaningful unless the iPSC state characteristics are also maintained. To demonstrate the maintenance of pluripotency, it was essential to test for the markers of undifferentiated iPSCs. At the end of the experiment, using flow cytometry we tested for SSEA4, OCT4, SOX2 and TRA-1-60 while a complimentary q-PCR assessment was conducted for NANOG, OCT4 and SOX2. The results of these experiments were used to model the effect of the additives on pluripotency. We optimized for the markers individually as shown in (Fig. 3A), since model results are non-confounding when optimization is performed for a singular response at a time. This is because that until proven that individual factors are not in regulatory conflict (i.e., SOX2, NANOG, or OCT4 being subject to differential control), single-response factor optimization is prudent. As we found, these factors are indeed subject to differential control.

Model optimizers for SOX2 and OCT4 had PEG as a major contributor with factor contributions (FC) of 18.72 and 53.88 respectively. The model for SOX2 also contained a significant contribution from Pluronic F68 (FC = 15.28 and 3% probability of failure). Optimization of NANOG had a contribution of PEG and Pluronic F68 with FC of 7.16 and 15.44 respectively, however the addition of HS and DS had the highest contribution for NANOG optimization with FC of 21.12 and 48.04 respectively. Validation using these optimizers are shown in stained images for the pluripotency markers (Fig. 3B). Of the pluripotency markers measured, OCT4 expression was most strongly affected by tested media additives (Fig. 3C). Multiple media additive combinations had unfavorable impact on the SOX2 and OCT4 expression. This indicated that certain media additive combinations are a critical attribute to maintaining the pluripotency in bioreactors. Coefficients for all flow and qPCR markers (Fig. 3D) demonstrated that DS had opposing impact on OCT4 (negative) and NANOG (positive) making it challenging to select additive inputs that would satisfy all pluripotent markers.

**Fig. 4:**
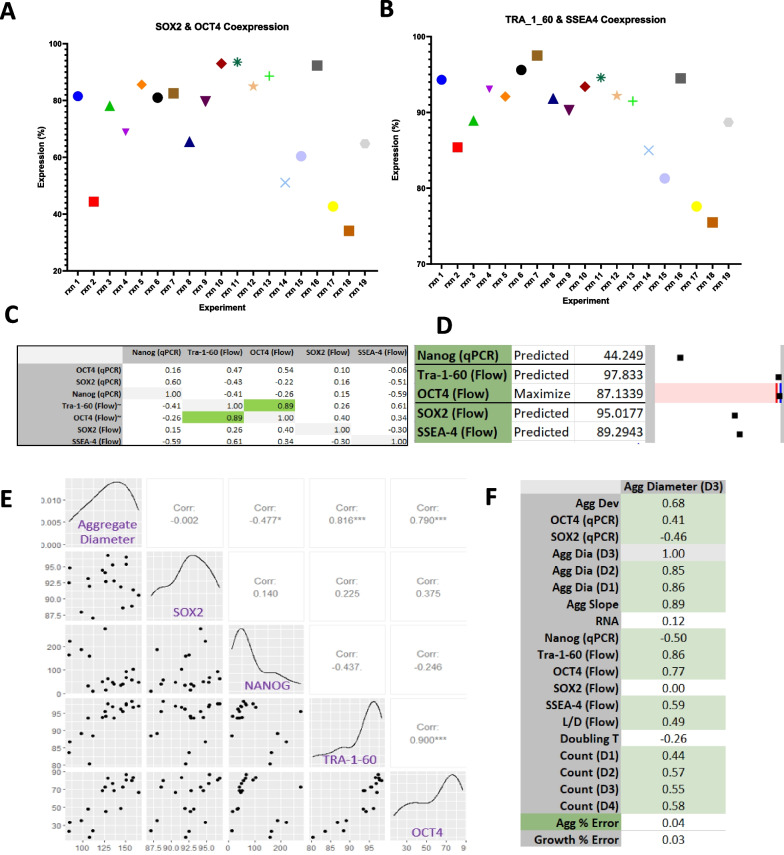
Modeling the coregulatory nature of pluripotency through set point and correlation analysis. **A** OCT4 and SOX2 co-expression comparison between all experiments. **B** SSEA4 and TRA-1-60 co-expression comparison between all experiments. **C** MODDE derived correlation coefficients for pluripotency markers. **D** Model for OCT4 maximization and predicted levels of other pluripotent markers. **E** Multi-scatter plot for the day 3 aggregate size diameter as compared to the core pluripotent markers SOX2, OCT4, TRA-1-60 and NANOG. This plot shows the correlation between all variables included. The strongest correlation seen to aggregate diameter is TRA-1-60 and OCT4 expression. **F** MODDE-based Correlation matrix between aggregate size and all other response variables measured

Co-expression of pluripotency intracellular markers (SOX2 and OCT4) with the extracellular markers (SSEA4 and TRA-1-60) was analyzed using flow cytometry (Additional file 1: Figs. S3 and S4). Comparative data showed co-expression of intracellular markers (Fig. 4A) and extracellular co-expression (Fig. 4B). Models demonstrated a strong correlation of 89% between OCT4 and TRA-1-60 expression (Fig. 4C). A correlation this strong was not observed between any of the other pluripotency markers. Using model optimizers for the maximization, and minimization, of OCT4 expression confirmed that TRA-1-60 was coregulated with OCT4 expression (Fig. 4D). This finding shows a direct predictive connection between OCT4 and TRA-1-60 suggesting a coregulatory relationship. The model showed additional correlation between these pluripotency markers and the response variable of aggregate size. Further validation of this correlation was evaluated using two statistical software tools, R (Fig. 4E) and MODDE (Fig. 4C, D, F). A positive correlation of 76–85% was observed between increased diameter size and OCT4/TRA-1-60 expression respectively (Fig. 4E, F). Whereas a negative correlation was observed between increased diameter size and SOX2 (-46%) and NANOG (-50%) expression (Fig. 4E, F). Correlation results from both statistical tools showed that there is a significant correlation between aggregate size change and the expression of pluripotency markers with a *P*-value < 0.05, therefore the observed correlation is unlikely to occur by chance alone.

### Controlling aggregate architecture using media additives

**Fig. 5:**
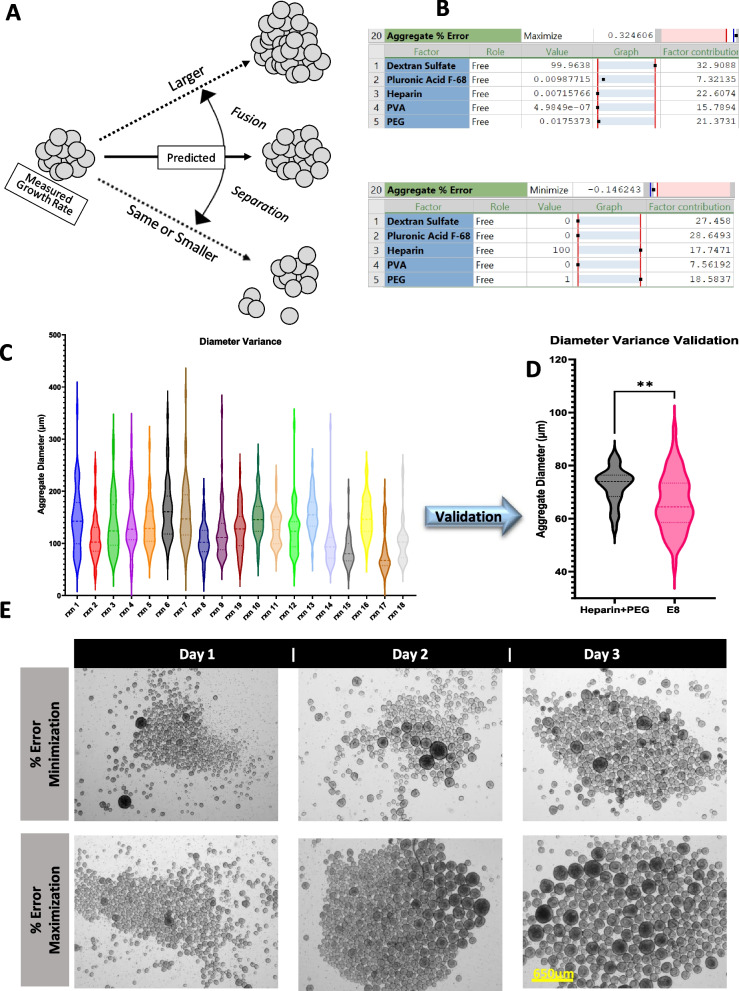
Optimizing for aggregate stability through predictive modeling. **A** A schematic showing expected aggregate growth in different scenarios. **B** Optimization for maximal or minimal ‘Aggregate % Error’ as indicated. **C** Violin plot of aggregate diameter distribution for all bioreactor runs. **D** Validation of aggregate stability optimizer as compared to E8. *P < 0.05, **P < 0.01, ***P < 0.001, ****P < 0.0001. **E** Daily aggregate pictures of experiments corresponding to aggregate % error minimization and maximization

Since aggregate size was demonstrated to impact pluripotency and its heterogeneity could cause inconsistencies in nutrients and oxygen intake by the cells, we sought to establish parameters qualifying the aggregate state. Ideally, optimal aggregate growth should occur through the formation of aggregates from single cells upon seeding, whereafter aggregate size growth should occur due to cell proliferation only. Large deviation in aggregate sizes could have two causes (1) unstable aggregates breaking apart due to shear forces and separate to become smaller or (2) aggregate fusion causing larger aggregates to form from the addition of smaller ones (Fig. 5A). Therefore, a parameter can be calculated and set for a desired range in an optimizer (Fig. 5B) using the data collected on cell proliferation and aggregate size throughout culture time. A violin plot of aggregate diameters in bioreactors on day 3 is shown (Fig. 5C) demonstrating that additives modify the distribution of the observed sizes. Aggregate sizes measured have a deviation and therefore modelling on average size could potentially mask that response output. To overcome this limitation of using the average aggregate size as a response variable, the average size was inputted in the model in addition to its corresponding standard deviation. Using the average aggregate size generated from day 1 from all reactions in the design with their corresponding growth rates, a predicted aggregate size was calculated for day 3 and compared to the observed size measured. We define this difference as “Aggregate % Error”. This is calculated by subtracting the predicted from the actual average size measured and divided by the observed. Using this derivative assay variables, it was then possible to obtain a combination of components that would predict stable size limiting, hereby limiting aggregate fusion (maximization) or aggregate instability (minimization) (Fig. 5B). The aggregate diameter deviation was calculated from aggregate diameter measurements of images taken on each culture day using ImageJ software. A known source of aggregate size increase is the adhesion of two or more aggregates into a large one [6]. It is desired that the formation and growth of aggregates is only a contribution of cell proliferation and not fusion between cell spheres. Processes such as changing the media, taking samples, or anything that removes bioreactors from the base, can create static cultures that lead to an environment promoting clump formation. Our optimizer for the target aggregate % error (Fig. 5B), revealed that the main contributors to improving aggregate stability and decreasing aggregate size deviation in culture were HS (21.71) and PEG (17.48). Inspection of the aggregate diameters and growth slope showed a decrease in this optimization which is desirable as it indicates control over aggregation growth. The cell state control was also evaluated at this setpoint. NANOG expression was close to maximum indicating pluripotency maintenance upon aggregate control. When looking at a comparison of the aggregate % error within the bioreactors, the bioreactor with the minimum error and deviation value was a reaction that contains DS, PVA, PEG and HS (Fig. 5D, E). Whereas the maximum % error was reaction 19 that was a center point containing all additives (Fig. 5E). Validation comparing the optimizer results to the control with the E8 medium with no additives (Fig. 5D) shows that the aggregate size spread was minimized with the addition of Heparin and PEG.

**Fig. 6:**
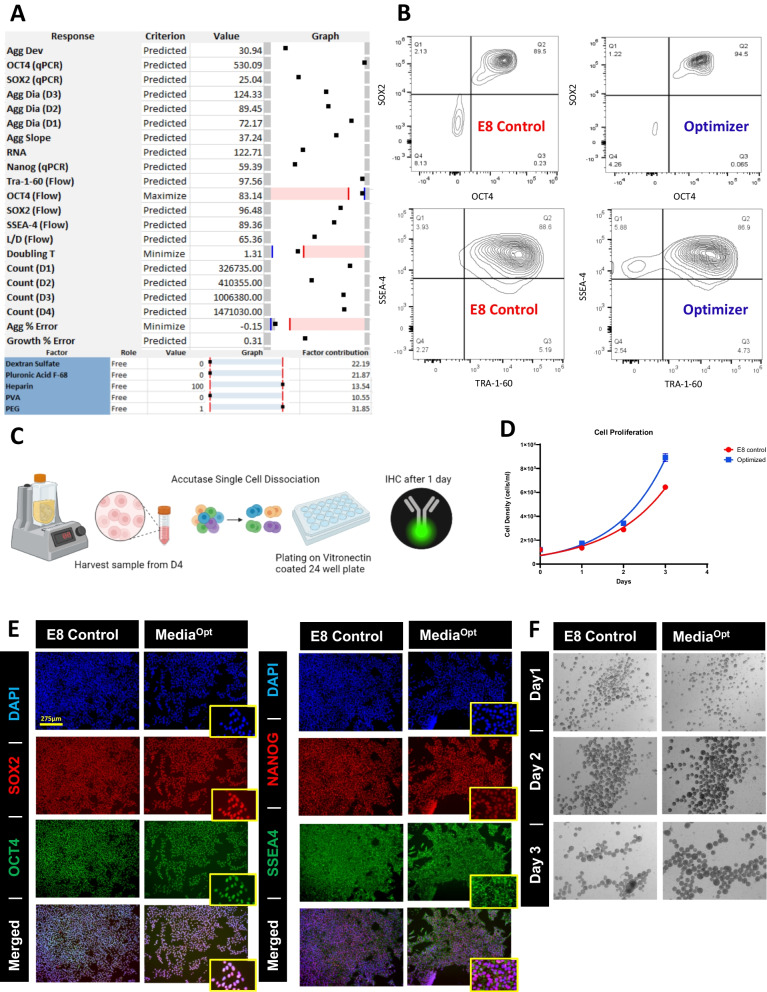
Improved aggregate stability through multilevel modeling of desired attributes. **A** MODDE optimizer for maximal OCT4 expression and doubling time with the minimal aggregate % error. **B** Flow cytometry-based pluripotency assessment of optimizer predicted conditions as compared to E8. **C** Schematic showing the method used for immunohistochemical staining of aggregate samples. Created with BioRender.com. **D** Growth rate assessment between optimizer conditions as compared to E8. *P < 0.05, **P < 0.01, ***P < 0.001, ****P < 0.0001. **E** Representative stains for pluripotent markers **F** Comparison of brightfield aggregates between optimizer conditions and E8

**Fig. 7:**
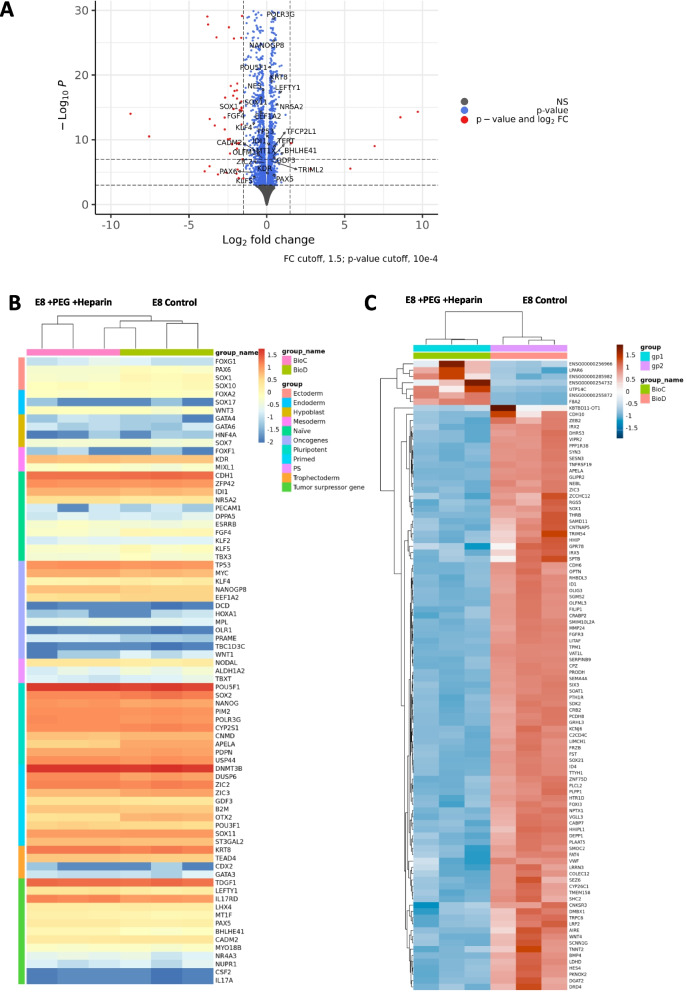
RNA-seq data. **A** Volcano plot showing the comparison of the optimized medium to the control medium (E8). **B** Heatmap comparison of selected genes evaluating pluripotency, differentiation, and other parameters. **C** Heatmap comparison of the top differentially expressed genes

Using an optimizer model, we determined the combination of factors that satisfies an overall desired output of growth rate, pluripotency, and aggregate stability (Fig. 6A), not sacrificing one over another. The results suggested that the combination of Heparin sodium salt and PEG can be added to maintain aggregate stability, pluripotency, and growth aspects simultaneously. Validation against an E8 control was done and maintenance of pluripotency (Fig. 6B, C), cell proliferation (Fig. 6D), and aggregate stability (Fig. 6E) were specifically addressed. After successful validation, the optimizer model was tested on an additional cell line RCRP005N as compared to NCRM-1 with a decreased bioreactor speed from 60 to 40 RPM. Both cell lines showed continuous aggregate growth (Additional file 1: Fig. S5A) even after passaging by reseeding 30 million cells as aggregates on the fourth day of culture. The cells maintained their pluripotency markers OCT4 and SOX2 (91.8% and 97.8% for RCRP5005N and 97.4% and 98.4% for NCRM-1 respectively) (Additional file 1: Fig. S5B). Cell growth rate was steady after serial passaging without dissociation (Additional file 1: Fig. S5C) with a doubling time of (1.222–1.434 days for RCRP5005N and 1.025–1.293 days for NCRM-1 before and after passaging respectively). To further evaluate the pluripotent nature of the sized controlled aggregates we next evaluated tri-lineage differentiation potential and karyotyping stability (Additional file 1: Fig. S6). The ability for trilineage differentiation was sustained in aggregates as evident through the selective activation of PAX6 and NES, the activation of FOXA2 and SOX17, and the activation of T and TNNT2 when challenged for ectodermal, endodermal, and mesodermal differentiation respectively. We complimented this pluripotent assessment through RNA sequencing. Volcano plots comparing the samples showed that few genes were responsive to the addition of PEG and HS (Fig. 7A). Evaluation of core pluripotent genes, showed that expression levels were similar between samples with a clear indication of a primed phenotype (Fig. 7B). In addition, we evaluated several well-known oncogenes [31] notable TP53, MYC, NANOGP8, EEF1A2 and KLF4 were expressed with similar transcript levels between samples, though KLF4 had low overall expression. Conversely, we examined tumor suppressor genes (TSR) [31] and several early lineage drivers. It was observed that a few genes indicative of forward differentiation were expressed with TSR genes TDGF1, LEFTY1 and IL17RC and the trophectodermal genes KRT8 and TEAD4 showing comparable expression levels. Expression of the primary primitive streak gene NODAL was observed at low transcript levels in both samples (Fig. 7B). To gain a better understanding of what transcripts were changing in expression in response to the addition of PEG and HS within bioreactors, a differential expression analysis was performed (Fig. 7C). Examining the top differentially expressed genes we noted that there were several genes involved in ectodermal differentiation and some key signaling pathway components that were significantly downregulated in response to the presence of PEG and HS. Down-regulated ectodermal genes included SOX1, OLIG3, LHX5 and OTX2 as well as some less known ones (Fig. 7C). The pathways most downregulated were the TGFβ family as suggested by BMP2 and BMP4 down regulation, though the previously mentioned levels of TDGF1, LEFTY1 and NODAL remained consistent to cultures lacking additives. Wnt family signaling components down regulated were Wnt4, FRZB, FZD5 and FRZ8. Also a decreased in retinoic acid signaling components were noted as indicated by decreased CYP26A1, CYP26C1, DHRS3 and CRABP2 transcript levels. These Results validated the robustness and repeatability of the predicted model. We conclude that the Aggregate stability variable can indirectly provide pluripotency maintenance and growth throughout the assay without impacting cell viability (Additional file 1: Fig. S5D). Therefore, this parameter can be regarded as a critical attribute that has a significant impact on iPSC cultures in a suspension environment.

## Discussion

Many efforts at upscaling hiPSC cultures utilize high-cost commercial medium with proprietary formulations. While adherent cultures predominantly use E8 medium, optimizing this culture medium to sustain a healthy and consistent aggregate culture is needed. Environmental and process stress such as hydrodynamic stress, single cell seeding, agitation and media changes are fundamentally different from adherent culture conditions. Here we empirically defined combinatorial media formulations that directly influence pluripotency, growth and aggregate stability using a five-dimensional DoE based approach. In this study, the optimal culture medium is defined as one that can maintain growth, aggregate stability, and pluripotency. This was determined to be E8 plus (0.1 µg/ml) Heparin and (10 mg/ml) PEG. These components positively influenced the majority of identified critical attributes (Fig. 6). Interactions between HS and PEG were well modeled, predicted, and enabled a better understanding of controlling aggregate stability. While together improving iPSC aggregate culturing, PEG and Heparin sodium salt have different effects on cellular aggregates. PEG is known to induce cell aggregation and promote the fusion of larger cell aggregates at higher concentrations (10% and above), while also capable of inhibiting aggregation in other applications at lower concentrations [16]. While PEG is also used in applications that mediates cell fusion to produce somatic cell hybrids, the fusion and mechanism targeted in this study is for aggregation only. Heparin sodium salt is known for its ability to interact with different growth factors such as FGF2 influencing cell behavior and pluripotency. Being able to detect this synergy within compounds that have a different effect on the culture reinforces the strength of the DoE approached used in this study. The only compounds used throughout this study that did not increase ‘aggregate % error’ were PEG and Heparin sodium salt. Modelling PEG indicated the overall effect was a reduced standard deviation of the aggregate diameter size, though no effect on doubling time was observed. The aggregate deviation and diameter growth rate increased slightly in the presence of PEG. While the addition of Heparin sodium salt did not show a strong impact on aggregate size or growth rate individually it was shown to have strong positive interaction in the presence of PEG.

Using DoE-based models provides several advantages over conventional one-factor-at-a-time methods. DoE modelling has embedded insights about model validity, reproducibility, fit (R2), and prediction precision (Q2) for each response variable measured allowing for an unbiased assessment of the overall model quality, all while identifying critical interactions. For example, all response variables measured throughout this series of experiments had an R2 value greater than 0.5, ensuring that models evaluated are highly significant. Models were also scrutinized for model validity and were only being considered significant when > 0.25, indicating an absence of outliers and transformation issues. As large-scale expansion of iPSCs and their derivatives become more common, DoE, as part of a Quality-by-Design strategy, should be used as a tool to evaluate multiple parameters impacting the overall cell quality and process stability. Embedding designs at increasing dimensional scales can be informative for the robust statistical analysis and unbiased optimization strategies needed when entering larger scale. Here, the modeling and design was performed within a low-shear reactor environment that is scalable. As there are multiple variables involved in large scale manufacturing and transferring adherent culture conditions/protocols to suspension culture, more efficient assays are needed that do not require long testing periods or sequential optimizations strategies lacking the ability to evaluate combinatorial interactions. Specifically, we were able to define unexpected combinations of media additives for controlling aggregate formation within a bioreactor system. We also obtained a rich data response space, where mathematical models included doubling time, aggregate deviation, aggregate growth slope, pluripotency markers and cell count measurements.

Using this methodical approach many novel insights into the compounds used in this study were discovered. DS, PVA and Pluronic F68 all had similar effects. Previous research showed that DS prevents cellular aggregation, controls aggregate size and growth properties [5, 26]. We could not replicate these observations. Set point analysis in MODDE can show how factors can vary depending on the selected setpoint and still meet the desired criteria and tolerances given. Set point analysis (Additional file 1: Fig. S7) showed that the largest deviation of ‘aggregate % error’ (Fig. 5A) was obtained using DS. In agreement with this observation modelling for maximal ‘aggregate % error’ resulted in a formulation predominantly containing DS (FC = 32.90) (Fig. 5B). Similarly, previous studies evaluating PVA effect on hiPSCs demonstrated that it promoted proliferation [23]. In contrast, set point analysis (Additional file 1: Fig. S7A) showed PVA impacts an increased aggregate diameter and the overall daily aggregate growth which is only desirable if these observed growth parameters occur solely due to cell proliferation. However, looking at the parameter of aggregate deviation (Additional file 1: Fig. S7A), indicates this PVA mediated increase was due to a heterogeneous effect on aggregate size. Collaborative evidence is provided by the fact that PVA had no effect on either growth rate or doubling time. A scenario repeated with Pluronic F68. Previous reports have shown that the inclusion of Pluronic F68 within a bioreactor increased the overall expansion capacity of hPSCs [15]. Here we show through a set point analysis that Pluronic F68 increased overall aggregate size and the deviation in the aggregate sizes (Additional file 1: Fig. S7A) again without effecting the aggregate growth slope, doubling time or cell concentration. Indicating again that this increase was due to fusion events between aggregates and not increased proliferation. An explanation for these discrepancies between previous studies is that others have compared individual reagents against controls lacking the additives, whereas in this study computer modelling allowed for the direct comparison of the compounds being evaluated. It should also be noted that Manstein et al. used Pluronic F68 so that RPM could be increased, an increased RPM would have countered the fusion effect. Overall, it is indicated here that fusion has a detrimental effect on cellular proliferation, potentially because as aggregates become larger nutrient availability is increasingly restrictive, actively inhibiting a proliferative state.

Essential 8 was used as the basal media for all reactions throughout this series of experiments and is therefore considered responsible for the general maintenance of pluripotency within the bioreactors. However, the compounds evaluated in this study did influence this maintenance. The provision of DS, Pluronic F68 or PVA all had an overall negative influence on pluripotent maintenance promoting the down regulation NANOG and SOX2 expression, though a slightly positive effect on OCT4 expression was shown. In contrast, PEG and Heparin sodium salt had a general positive effect on pluripotent maintenance showing a positive correlation to OCT4 and NANOG expression, while having differential influence on SOX2 expression with Heparin sodium salt decreasing its expression and PEG increasing SOX2 levels (Additional file 1: Fig. S7A). In general, we could not find strong correlations between the core pluripotency markers OCT4, SOX2, and NANOG (Fig. 4C). A possible explanation is that they have divergent roles functioning as master regulators of lineage-specific differentiation as others have suggested. Previous studies have shown that the over expression of OCT4 leads to endodermal and mesodermal fates [32], whereas the overexpression of SOX2 generates ectodermal fate [33] suggesting that driving the maximal expression of either would result in the down regulation of the other. Modelling for minimal or maximal OCT4 expression indicated that the only pluripotent marker assayed within this study that followed OCT4 expression was TRA-1-60 (a positive correlation of 0.89), though notably aggregate diameter correlated with both pluripotency markers. This model was confirmed using R Software (Fig. 4E), which indicated a strong correlation between the increase of the diameter size and the expression of OCT4 and TRA-1-60 markers (0.790, 0.816) with a high significance level (*P*-value < 000.1). This suggests that aggregate size could be used to influence differentiation potential, specifically here that endoderm differentiation may benefit from a population of larger aggregate. This could also explain why compounds that promoted fusion selectively lose SOX2 and NANOG expression while showing a small increase in OCT4 expression.

One of the major efforts during this study was the development of quantification methods for aggregate stability with the overall goal of limiting aggregate fusion. Controlling aggregate size allows for a decrease in agitation rate required in the bioreactor and therefore reduces sheer stress as described in recent publication from Borys, Breanna S et al. [34]. Measurements of aggregate size deviation (range of sizes) could determine the overall homogeneity of aggregate size but could not directly inform on fusion since differential growth rates between aggregates and aggregate fractioning due to shear stress are alternative explanations. So, to better define what was occurring on the individual aggregate level a method for quantifying this aggerate parameter was developed. Resting on a theoretical estimation of the rate aggregate size should change according to growth rates observed, we directly compared this to measured size changes observed within the bioreactors. This was defined as ‘aggregate % error’. We found that by modelling using this parameter we could gain a better control over the size and overall homogeneity of the aggregates within bioreactors. Models setting the ‘aggregate % error’ to the predicted diameter (theoretical size based on doubling time) would define conditions that lacked fusion but in which aggregates were stable enough to resist shear forces. While this model proved useful in measuring overall aggregate stability there are inherent limitations to it. The mathematical model assumes cell size uniformity, a parameter likely to change based on the density of the individual aggregates or differentiation state of the cell culture. There is also an assumption based on empirical observations and simulation experiments that the cells are randomly packed. An alternative method for estimating aggregate stability would have been directly measuring aggregate numbers daily, decreasing aggregate number with an increasing aggregate size could directly indicate fusion. Here we show that models quantifying aggregate behavior based on calculations of theoretical predictions as compared to empirical results are crucial for the identification and understanding of the complexities in a biological system. Having predictive models allows for formulating a hypothesis about the underlying process mechanisms and therefore facilitates optimization by identifying key factors and variables that bring the model to desired results.

## Conclusion

For each model the relationship between additive/response variable could be analyzed and optimized towards desirability criteria. In all desirability contexts, process capability (Cpk) as a measurement of process robustness was favorable. Optimal conditions for maximal cell growth required combinatorial additive use. Similarly, optimization of the maintenance of pluripotency, as based on markers OCT4, NANOG, SOX2 was also dependent on combinatorial signaling. Based on measurements of aggregate size and size variance, optimization could be performed to increase aggregate homogeneity. In all cases, underlying models included multiple interaction terms, revealing the criticality of simultaneous testing using combinatorial designs. It was demonstrated that the optimal culture medium with the combinatorial effects of Polyethylene Glycol and Heparin Sodium Salt could maintain growth, pluripotency and control aggregate stability by limiting fusion events between aggregates in suspension cultures. We conclude that DoE-based interaction testing performed within a manufacturing-relevant environment allows for process understanding of the biomanufacturing process. The method identifies critical process parameters; their interacting criticality, while returning a deep process understanding.

### Supplementary Information

**Additional file 1**. Table of the complete media formulations used throughout the DoE design.

## Data Availability

Requests for further information or more detailed protocols should be directed to and will be fulfilled by the corresponding author. This study did not generate new unique reagents. All the RNA-Seq data are deposited in NCBI’s Gene Expression Omnibus (GEO) dataset with the accession number GSE264081. The data that support the findings of this study are available on request.

## Abbreviations

HS: Heparin sodium salt
PEG: Polyethylene glycol
PVA: Poly (vinyl alcohol)
PA: Pluronic F68
DS: Dextran sulfate
E8: Essential 8
DMSO: Dimethyl Sulfoxide
DoE: Design of experiments
3D: Three-dimensional
cGMP: Current Good manufacturing practice
hiPSC: Human Induced pluripotent stem cell
PBS: Phosphate buffer solution
q-PCR: Quantitative PCR
RPM: Revolution per minute
ROCK: Rho-Kinase
TSR: Tumor suppressor genes

## Declarations

### Artificial intelligence

The authors declare that artificial intelligence is not used in this study.

## References

[1] Takahashi K, Yamanaka S (2006). Induction of pluripotent stem cells from mouse embryonic and adult fibroblast cultures by defined factors. Cell.

[2] Fan Y, Wu J, Ashok P, Hsiung M, Tzanakakis ES (2015). Production of human pluripotent stem cell therapeutics under defined xeno-free conditions: progress and challenges. Stem Cell Rev Rep.

[3] Deinsberger J, Reisinger D, Weber B (2020). Global trends in clinical trials involving pluripotent stem cells: a systematic multi-database analysis. NPJ Regen Med.

[4] Colter J, Murari K, Biernaskie J, Kallos MS (2021). Induced pluripotency in the context of stem cell expansion bioprocess development, optimization, and manufacturing: a roadmap to the clinic. NPJ Regen Med.

[5] Nogueira DES, Rodrigues CAV, Carvalho MS, Miranda CC, Hashimura Y, Jung S (2019). Strategies for the expansion of human induced pluripotent stem cells as aggregates in single-use Vertical-Wheel™ bioreactors. J Biol Eng.

[6] Otsuji TG, Bin J, Yoshimura A, Tomura M, Tateyama D, Minami I (2014). A 3D sphere culture system containing functional polymers for large-scale human pluripotent stem cell production. Stem Cell Rep.

[7] Abbasalizadeh S, Larijani MR, Samadian A, Baharvand H (2012). Bioprocess development for mass production of size-controlled human pluripotent stem cell aggregates in stirred suspension bioreactor. Tissue Eng Part C Methods.

[8] Liu M (2013). Engineering stem cell niches in bioreactors. World J Stem Cells.

[9] Teramura T, Takehara T, Onodera Y, Nakagawa K, Hamanishi C, Fukuda K (2012). Mechanical stimulation of cyclic tensile strain induces reduction of pluripotent related gene expressions via activation of Rho/ROCK and subsequent decreasing of AKT phosphorylation in human induced pluripotent stem cells. Biochem Biophys Res Commun.

[10] Saha S, Ji L, de Pablo JJ, Palecek SP (2008). TGFβ/activin/nodal pathway in inhibition of human embryonic stem cell differentiation by mechanical strain. Biophys J.

[11] Kim YM, Kang YG, Park SH, Han M-K, Kim JH, Shin JW (2017). Effects of mechanical stimulation on the reprogramming of somatic cells into human-induced pluripotent stem cells. Stem Cell Res Ther.

[12] Goetzke R, Sechi A, De Laporte L, Neuss S, Wagner W (2018). Why the impact of mechanical stimuli on stem cells remains a challenge. Cell Mol Life Sci.

[13] Xu C, Rosler E, Jiang J, Lebkowski JS, Gold JD, O’Sullivan C (2005). Basic fibroblast growth factor supports undifferentiated human embryonic stem cell growth without conditioned medium. Stem Cells.

[14] Kuo H-H, Gao X, DeKeyser J-M, Fetterman KA, Pinheiro EA, Weddle CJ (2020). Negligible-cost and weekend-free chemically defined human iPSC culture. Stem Cell Rep.

[15] Manstein F, Ullmann K, Kropp C, Halloin C, Triebert W, Franke A (2021). High density bioprocessing of human pluripotent stem cells by metabolic control and in silico modeling. Stem Cells Transl Med.

[16] Castellanos IJ, Crespo R, Griebenow K (2003). Poly(ethylene glycol) as stabilizer and emulsifying agent: a novel stabilization approach preventing aggregation and inactivation of proteins upon encapsulation in bioerodible polyester microspheres. J Control Release Off J Control Release Soc.

[17] Chen G, Gulbranson DR, Hou Z, Bolin JM, Ruotti V, Probasco MD (2011). Chemically defined conditions for human iPSC derivation and culture. Nat Methods.

[18] Wilken S, Guerra RE, Levine D, Chaikin PM (2021). Random close packing as a dynamical phase transition. Phys Rev Lett.

[19] Bukys MA, Mihas A, Finney K, Sears K, Trivedi D, Wang Y (2020). High-dimensional design-of-experiments extracts small-molecule-only induction conditions for dorsal pancreatic endoderm from pluripotency. iScience..

[20] Hu W, Rathman JJ, Chalmers JJ (2008). An investigation of small-molecule surfactants to potentially replace pluronic F-68 for reducing bubble-associated cell damage. Biotechnol Bioeng.

[21] Koski A, Yim K, Shivkumar S (2004). Effect of molecular weight on fibrous PVA produced by electrospinning. Mater Lett.

[22] Wilkinson AC, Ishida R, Kikuchi M, Sudo K, Morita M, Crisostomo RV (2019). Long-term ex vivo haematopoietic-stem-cell expansion allows nonconditioned transplantation. Nature.

[23] Tang X, Wu H, Xie J, Wang N, Chen Q, Zhong Z (2021). The combination of dextran sulphate and polyvinyl alcohol prevents excess aggregation and promotes proliferation of pluripotent stem cells in suspension culture. Cell Prolif..

[24] Braam SR, Zeinstra L, Litjens S, Ward-van Oostwaard D, van den Brink S, van Laake L (2008). Recombinant vitronectin is a functionally defined substrate that supports human embryonic stem cell self-renewal via αVβ5 integrin. Stem Cells.

[25] Furue MK, Na J, Jackson JP, Okamoto T, Jones M, Baker D (2008). Heparin promotes the growth of human embryonic stem cells in a defined serum-free medium. Proc Natl Acad Sci.

[26] Lipsitz YY, Tonge PD, Zandstra PW (2018). Chemically controlled aggregation of pluripotent stem cells. Biotechnol Bioeng.

[27] Chisti Y (2000). Animal-cell damage in sparged bioreactors. Trends Biotechnol.

[28] Papoutsakis E (1991). Media additives for protecting freely suspended animal cells against agitation and aeration damage. Trends Biotechnol.

[29] Goldblum S, Bae YK, Hink WF, Chalmers J (1990). Protective effect of methylcellulose and other polymers on insect cells subjected to laminar shear stress. Biotechnol Prog.

[30] Kane VE (1986). Process capability indices. J Qual Technol.

[31] Ou M, Zhao M, Li C, Tang D, Xu Y, Dai W (2021). Single-cell sequencing reveals the potential oncogenic expression atlas of human iPSC-derived cardiomyocytes. Biol Open..

[32] Niwa H, Miyazaki J, Smith AG (2000). Quantitative expression of Oct-3/4 defines differentiation, dedifferentiation or self-renewal of ES cells. Nat Genet.

[33] Kopp JL, Ormsbee BD, Desler M, Rizzino A (2008). Small increases in the level of Sox2 trigger the differentiation of mouse embryonic stem cells. Stem Cells.

[34] Borys BS, Dang T, So T, Rohani L, Revay T, Walsh T (2021). Overcoming bioprocess bottlenecks in the large-scale expansion of high-quality hiPSC aggregates in vertical-wheel stirred suspension bioreactors. Stem Cell Res Ther.

