# Supplementary material for: Addressing bioreactor hiPSC aggregate stability, maintenance and scaleup challenges using a design of experiment approach
Source: Stem Cell Res Ther. 2024 Jul 2;15:191. doi: 10.1186/s13287-024-03802-4 (PMC11218057; doi:10.1186/s13287-024-03802-4)
Supplement: Supplementary file 1 — Additional file 1. Table of the complete media formulations used throughout the DoE design. [file 13287_2024_3802_MOESM1_ESM.docx]

**Supplemental Figures and Legends:**

**A**


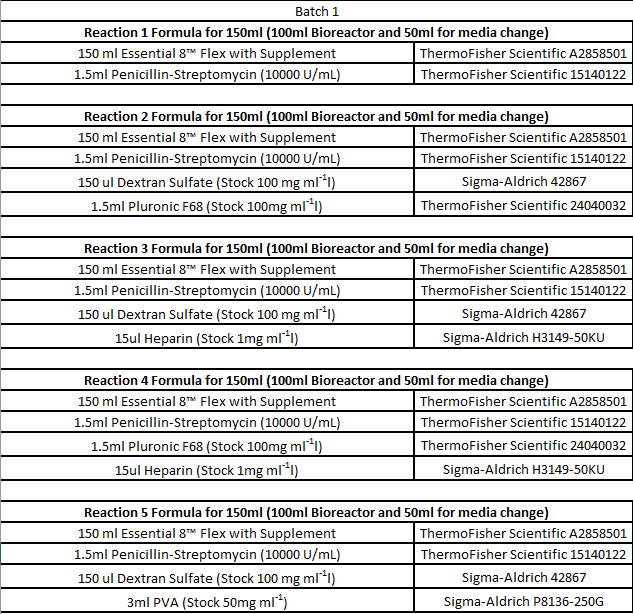


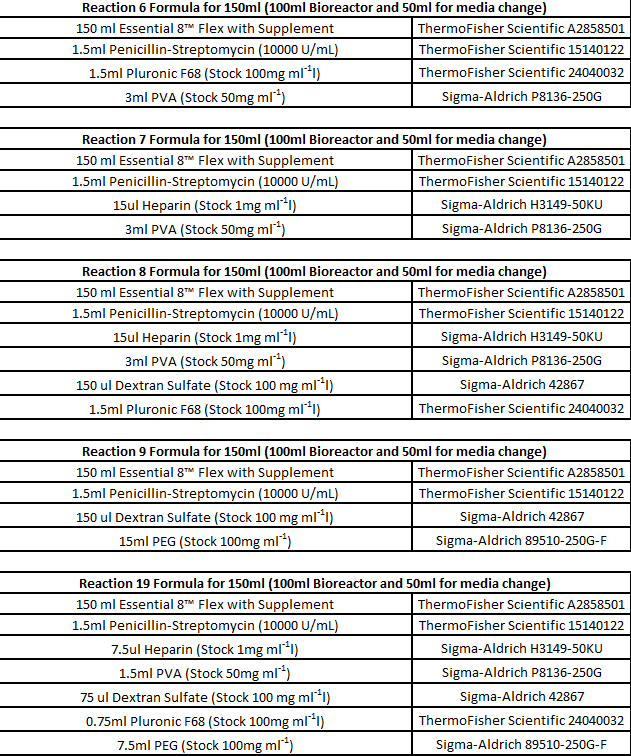


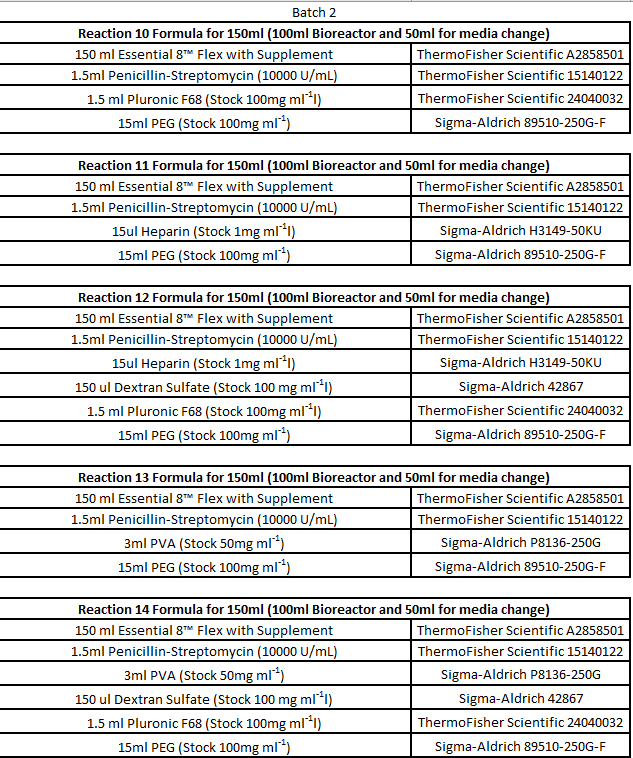


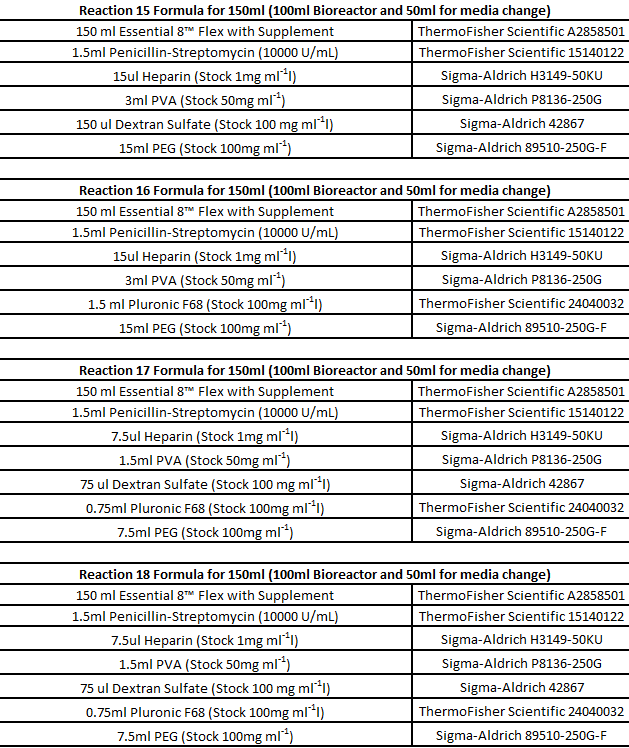


**
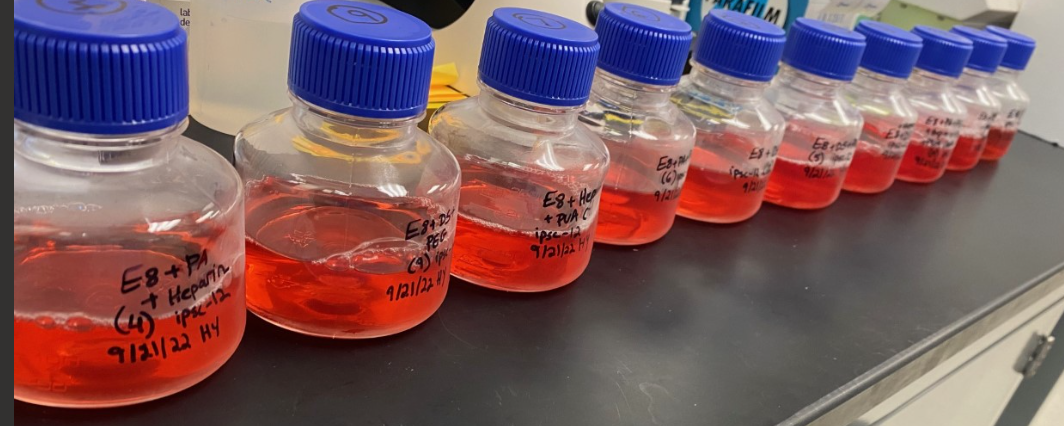
**

**B**

**
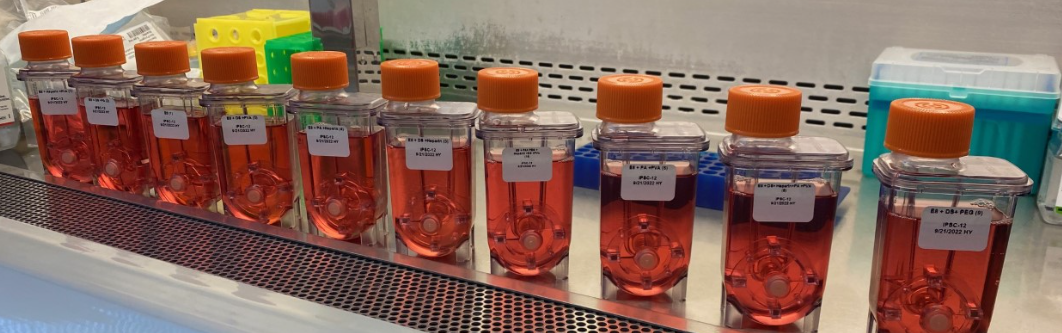
**

**Figure S1: Reaction Additives and Experimental Set Up**

1. Reaction additives for all bioreactors in the experiment
2. Media and bioreactor set up for the first batch of experiments.

**
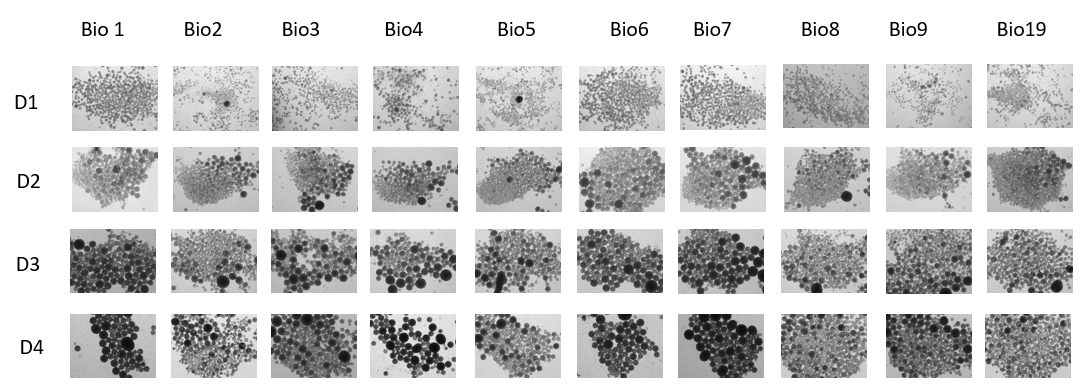

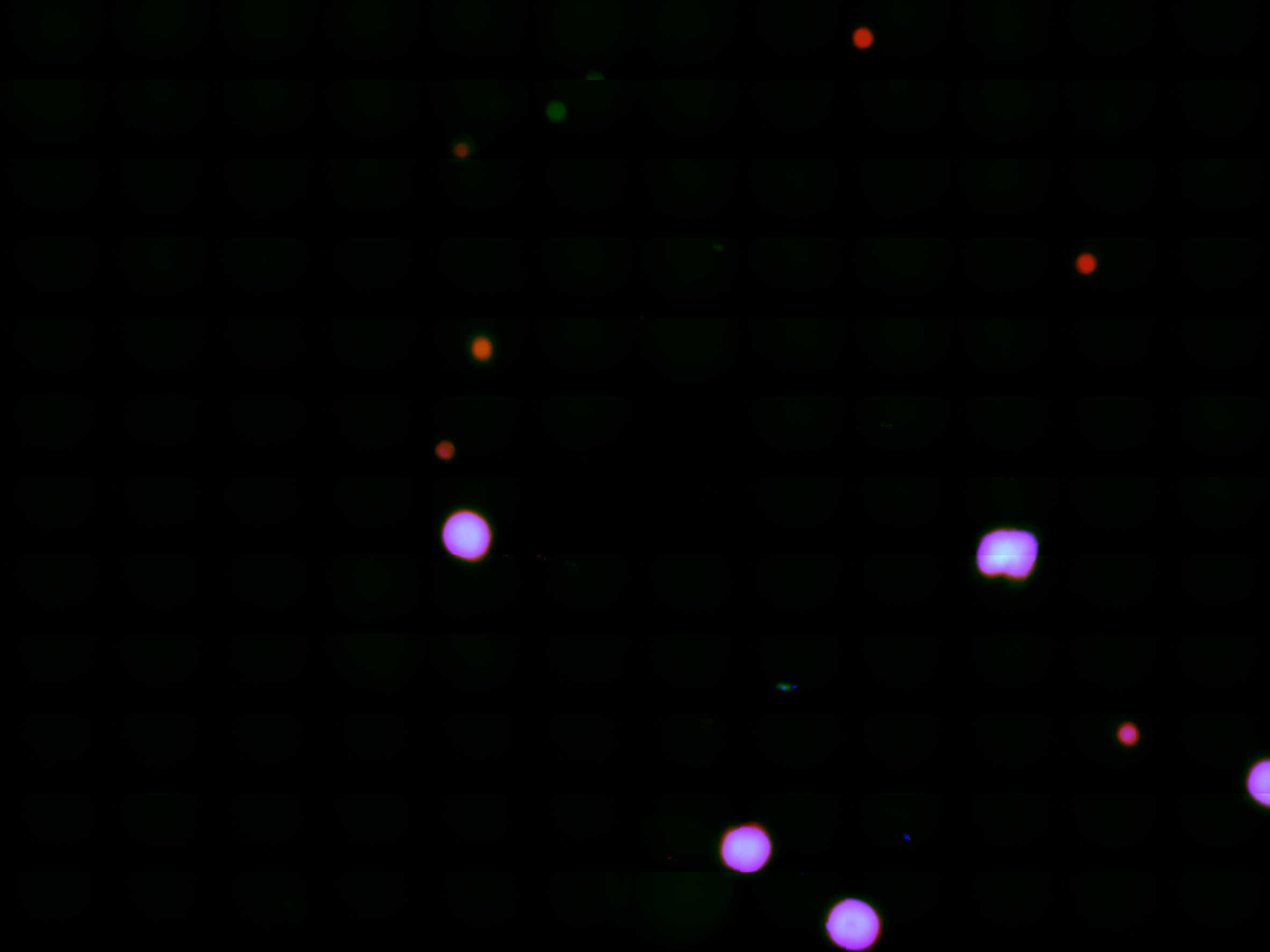

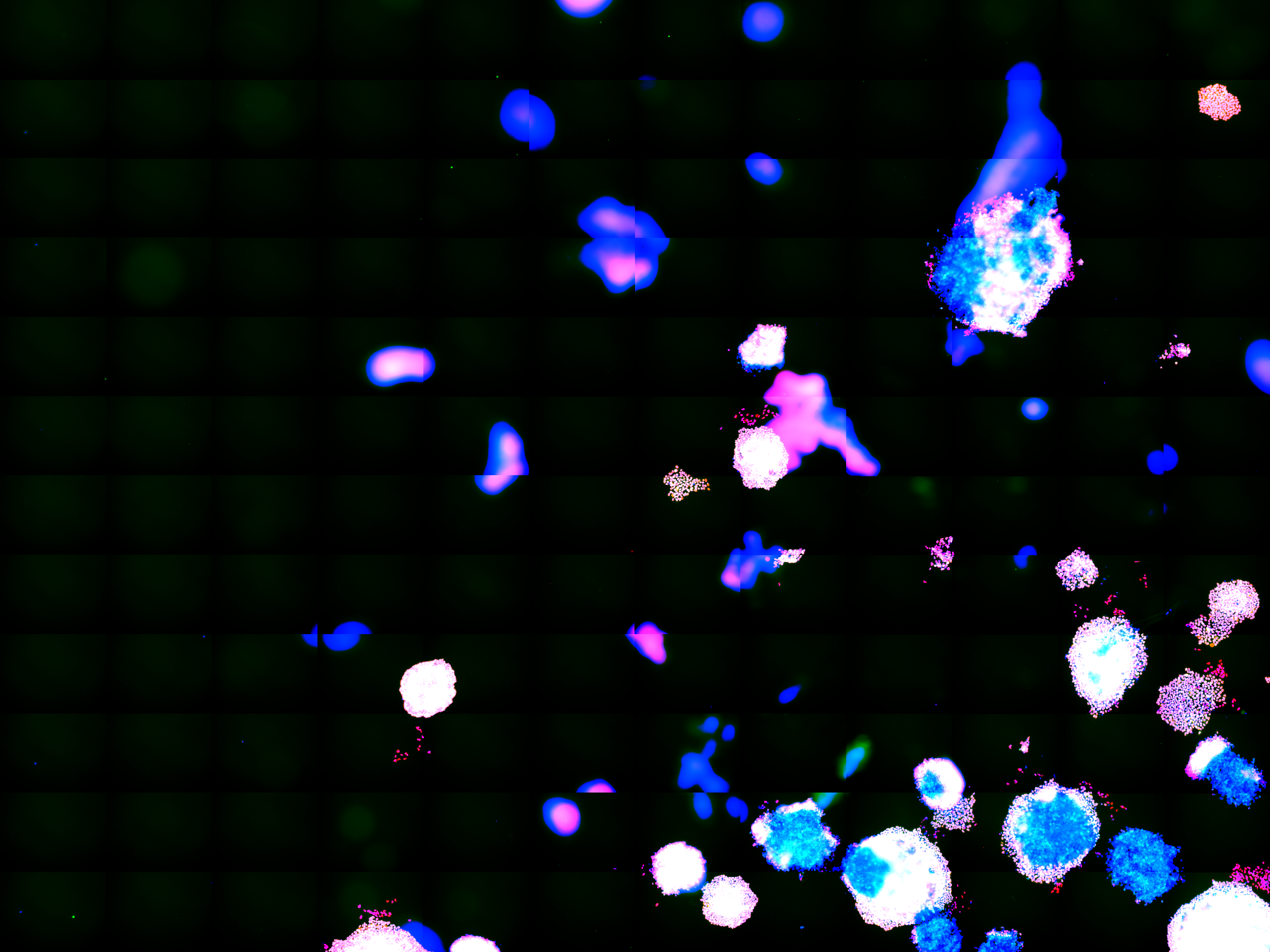

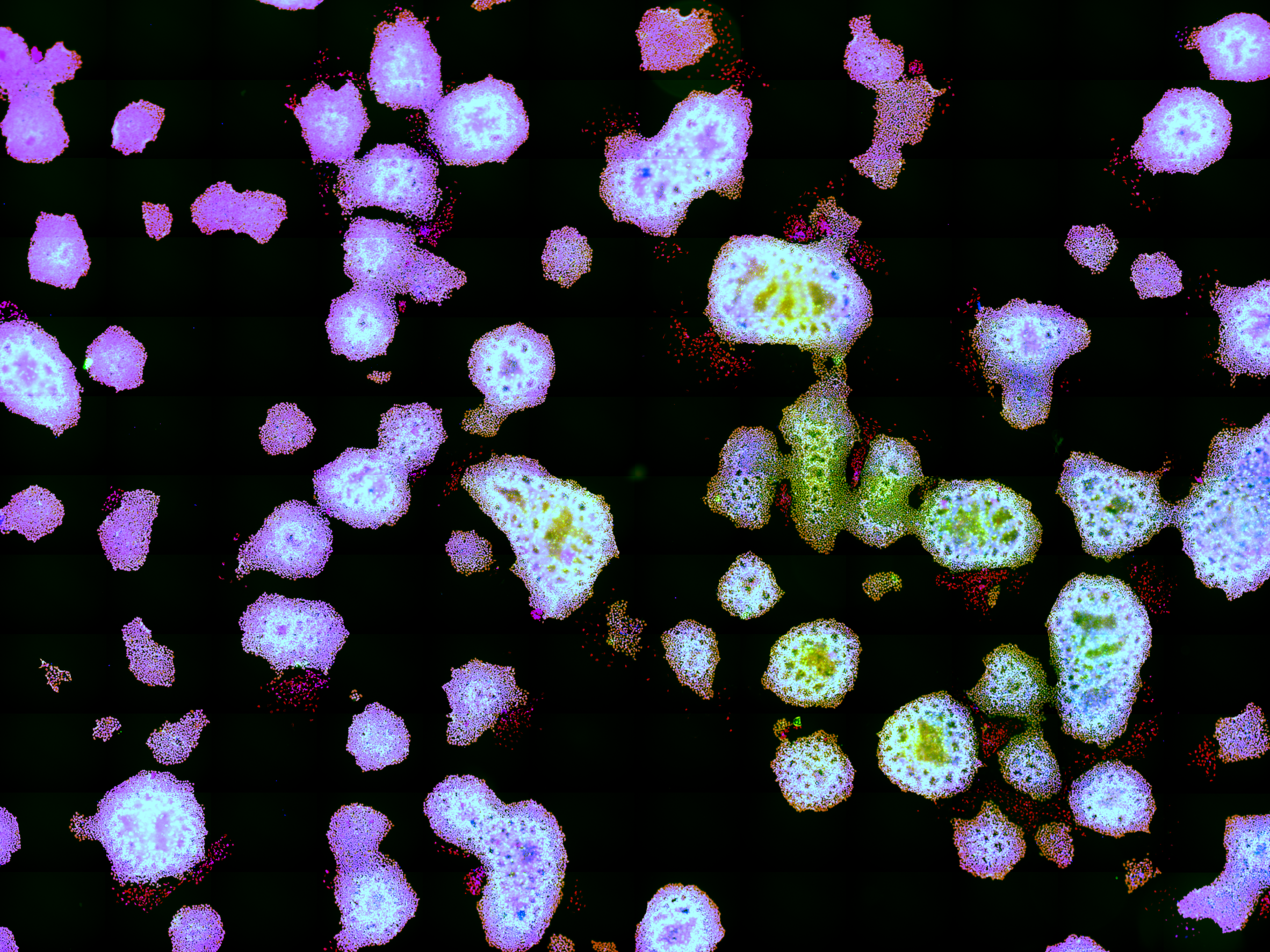

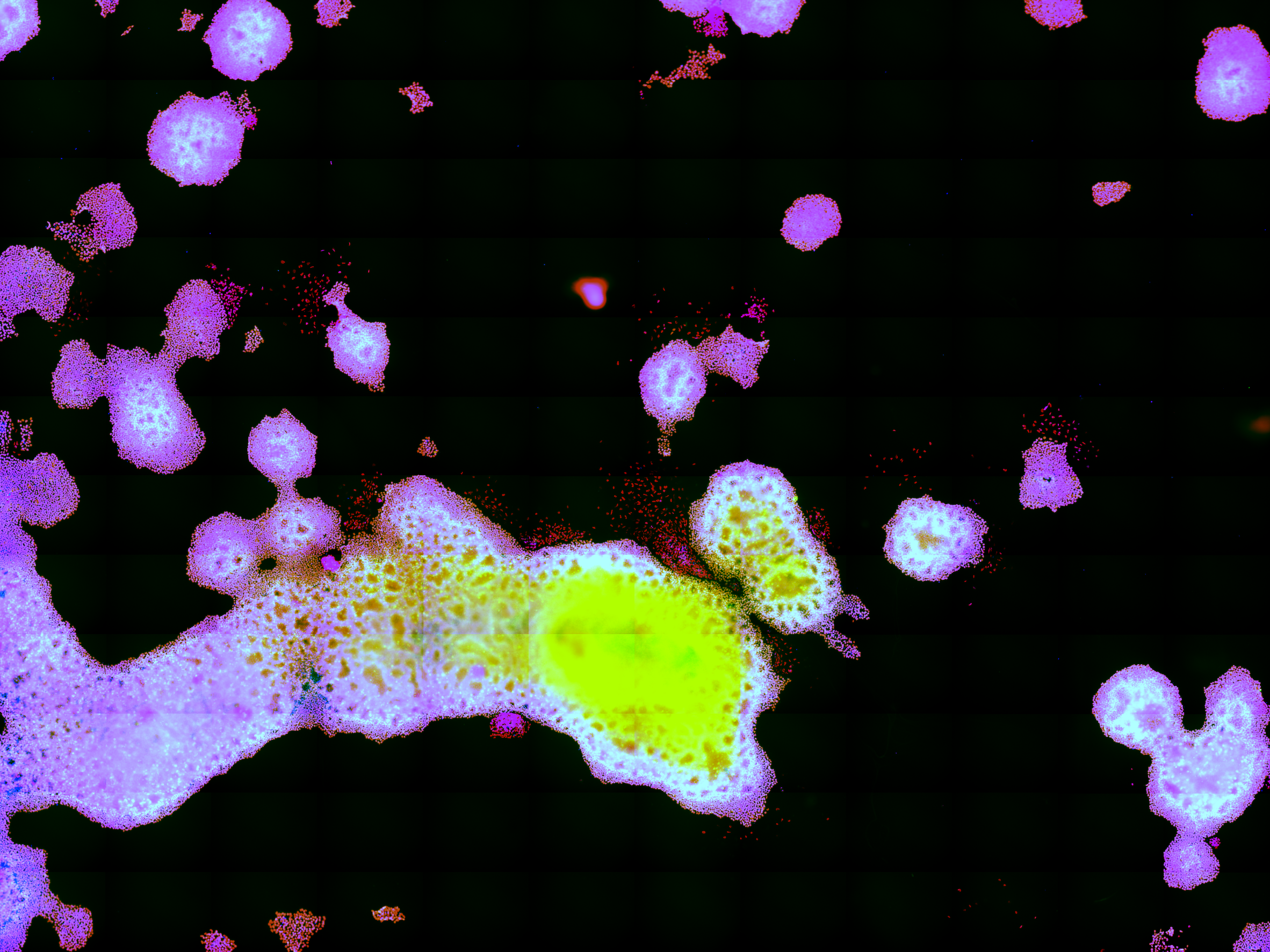

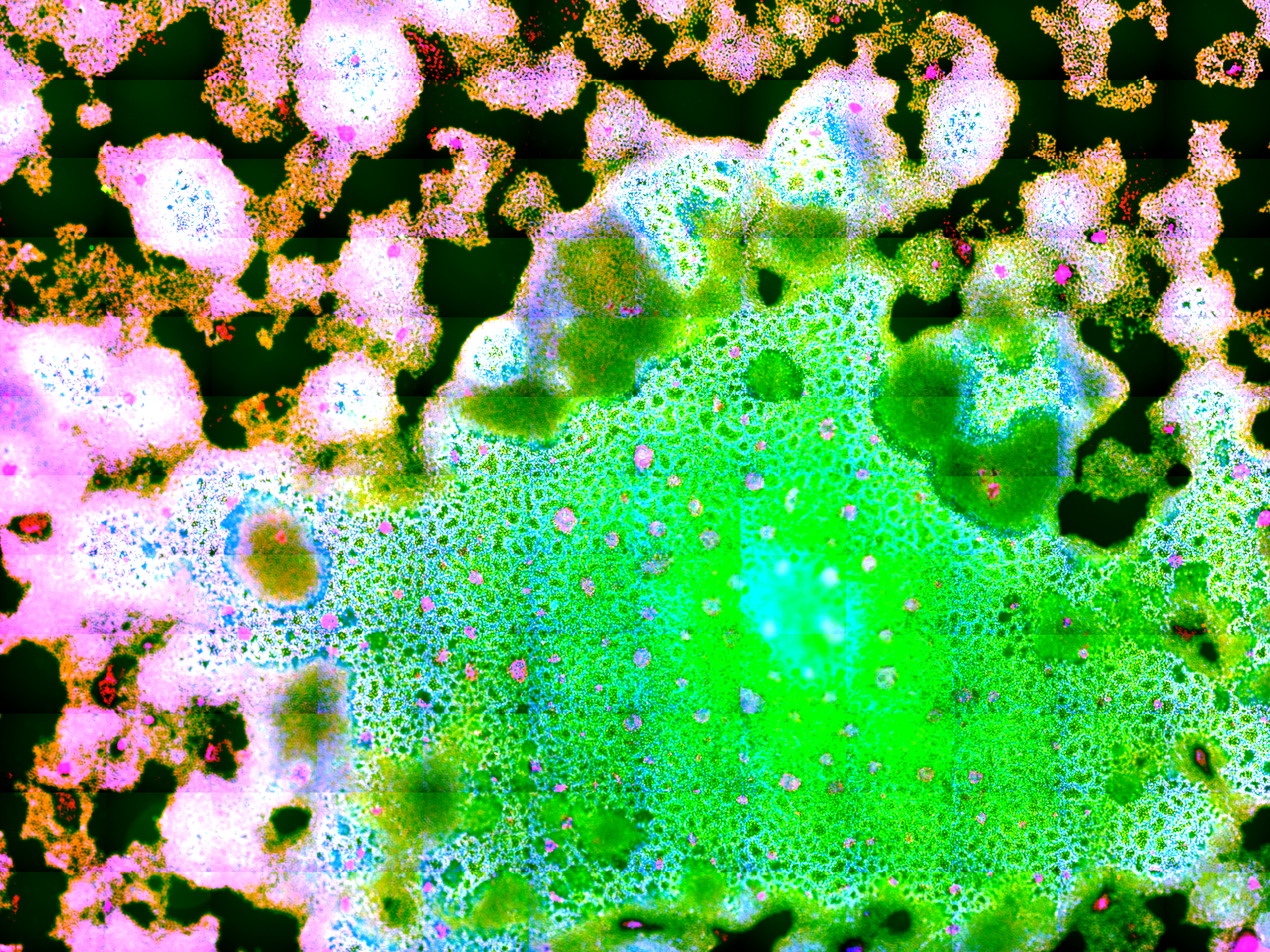

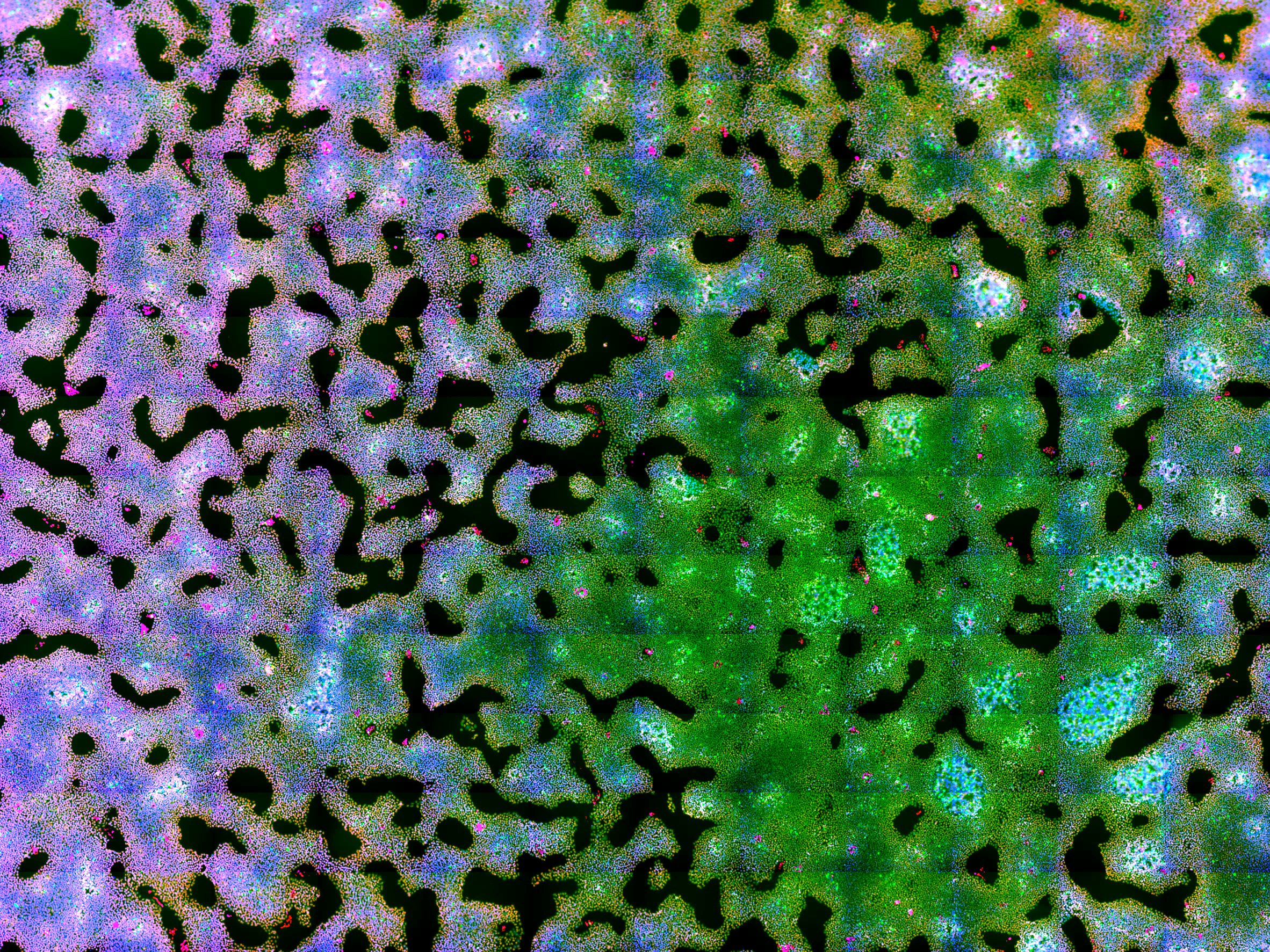

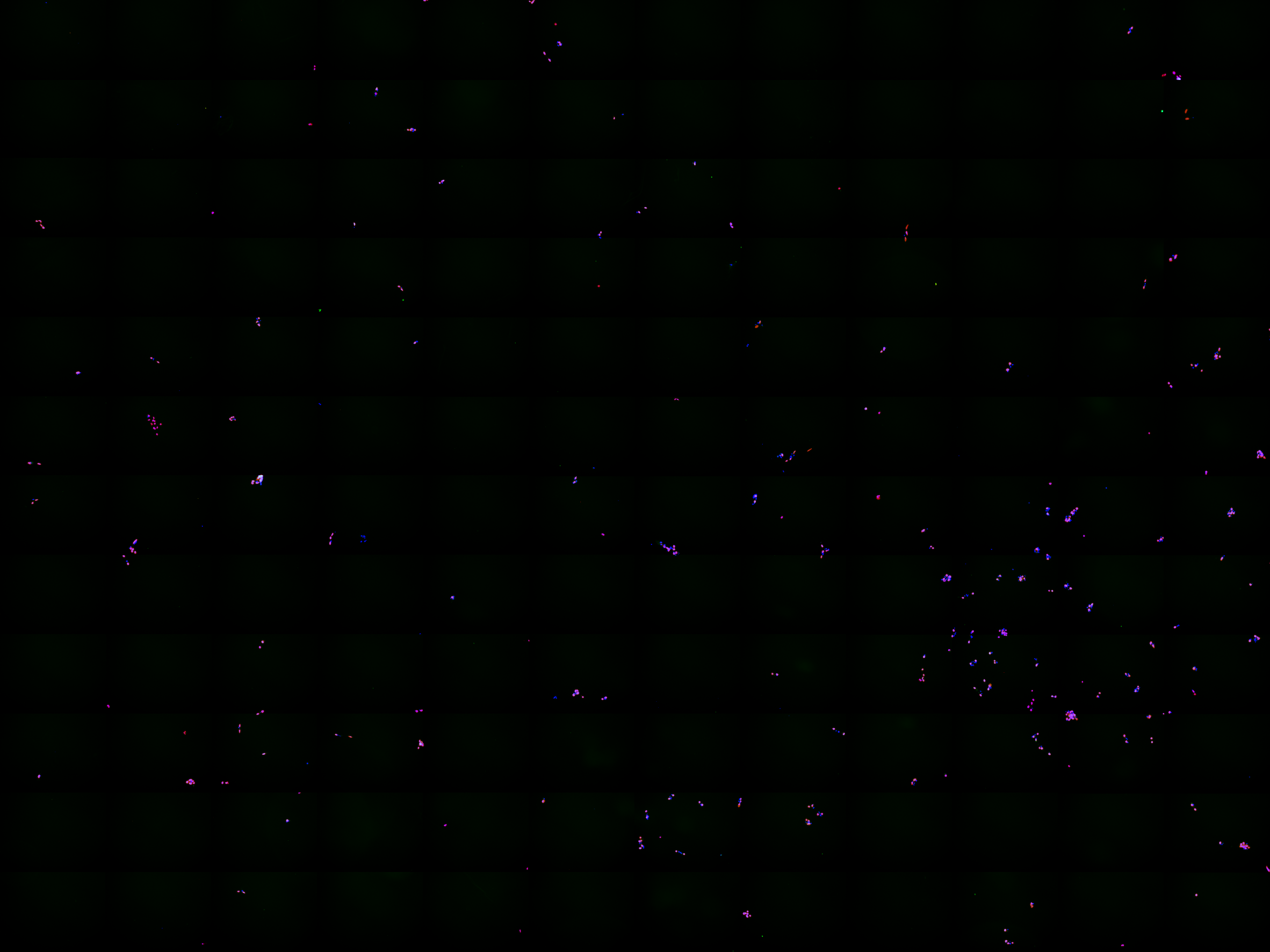

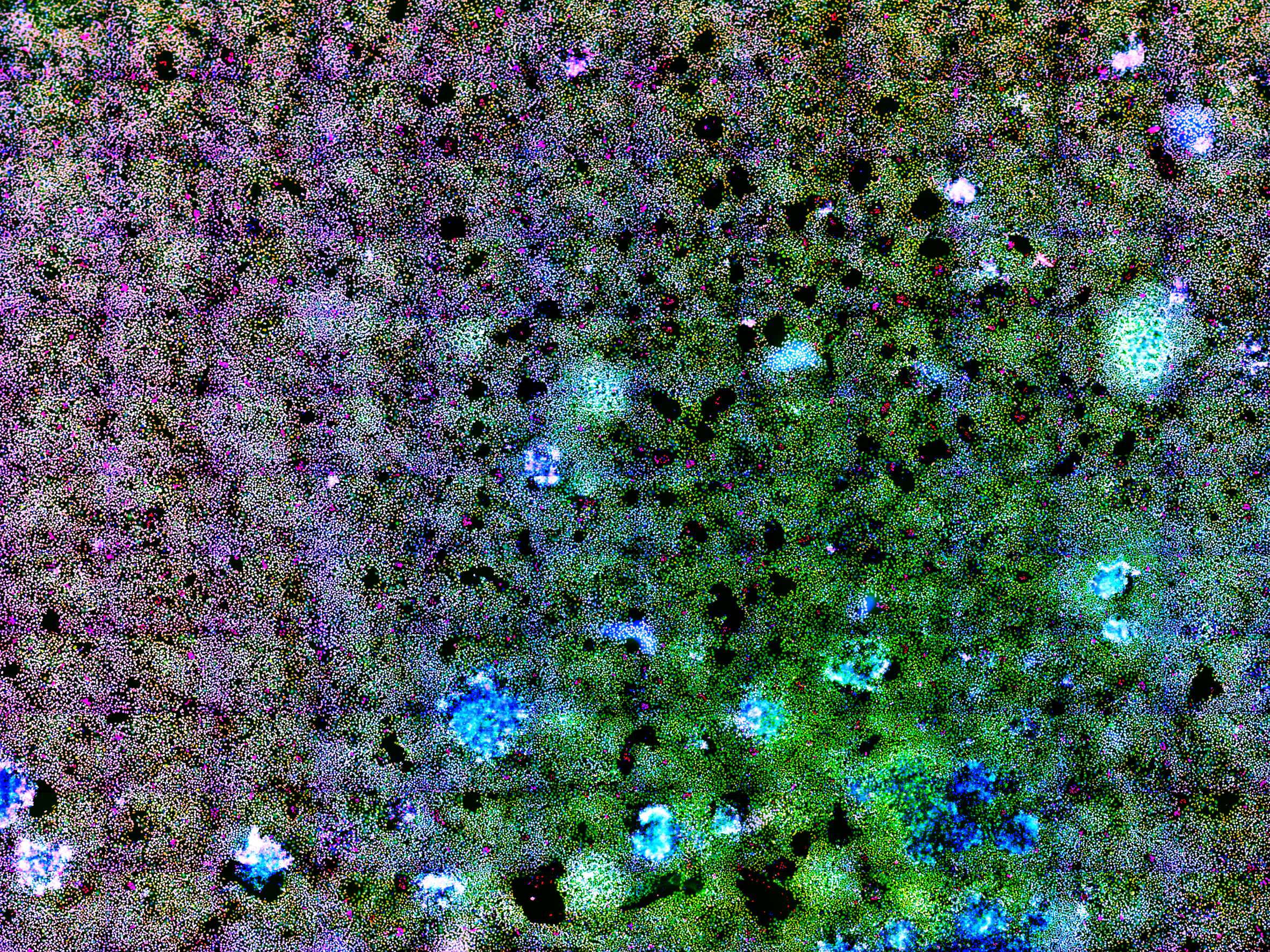

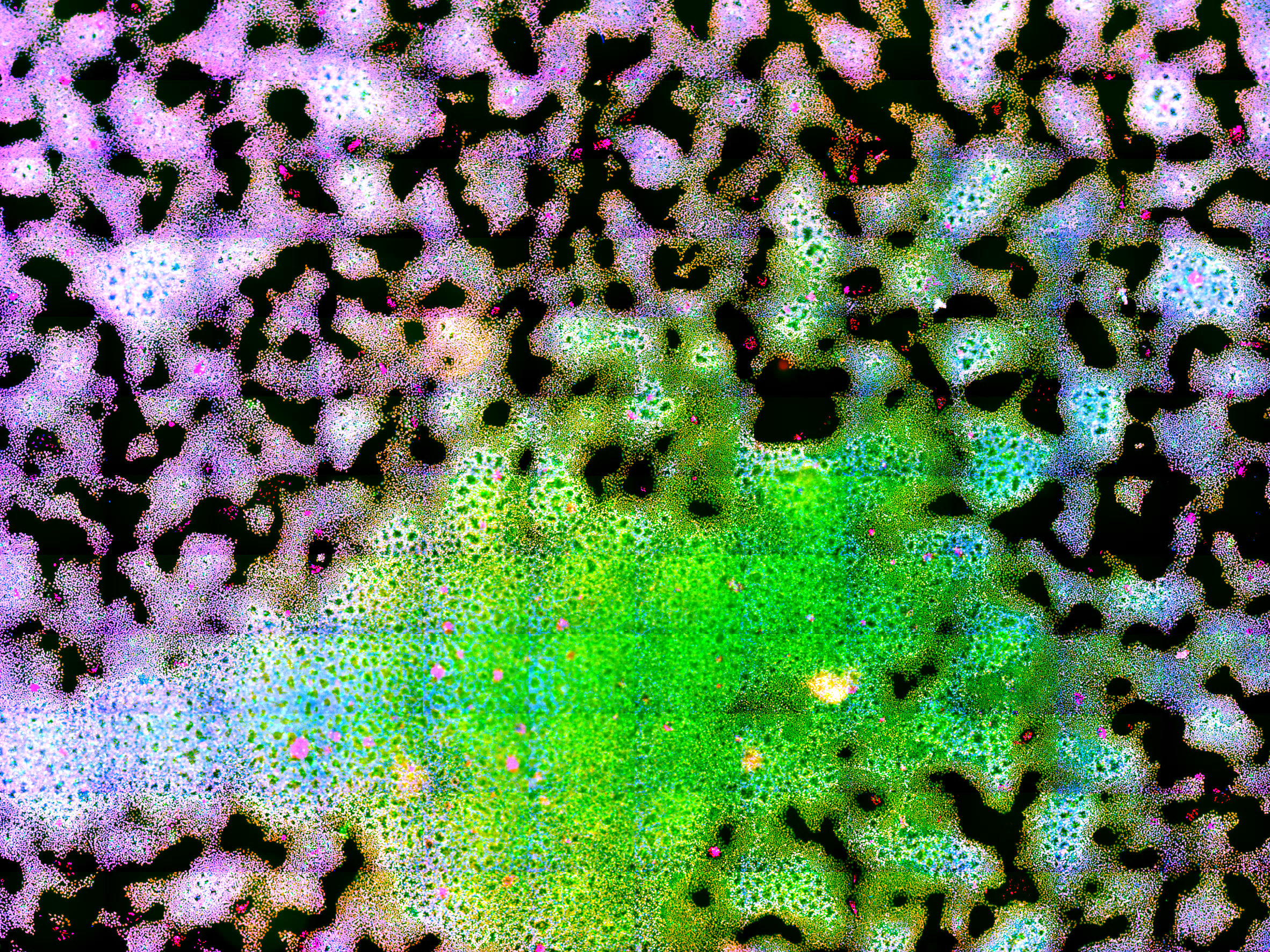

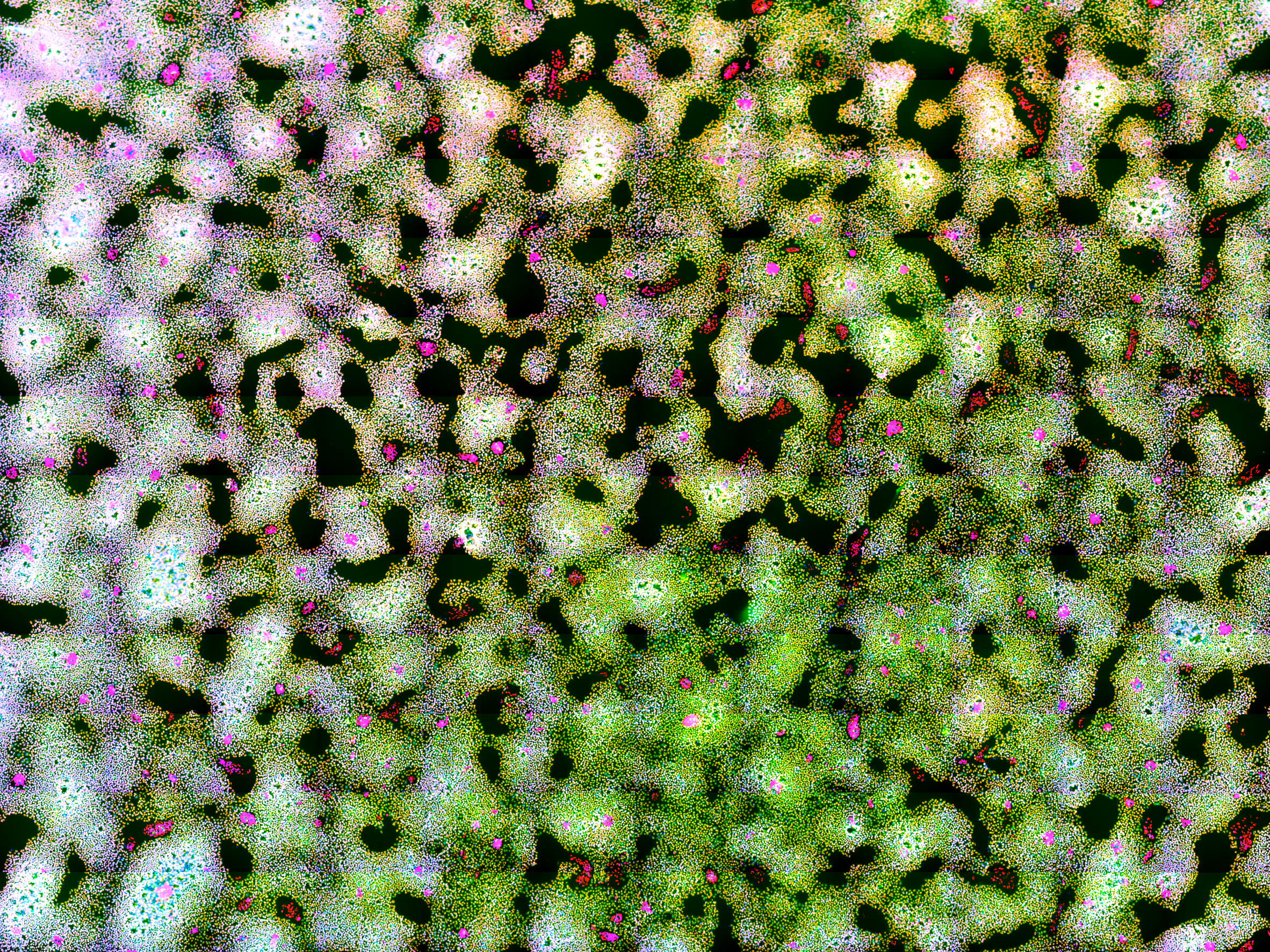
Suppl****emental Figures and Legends:**

**E**

**C**

**rxn1 rnx2 rxn3 rxn4 rxn5 rxn6 rxn7 rxn8 rxn9 rxn19**

Batch 1

**C**

*Aggregates*

Day 3

Day 2

Day 4

Day 1

Day 0

Day 3

Day 2

Day 4

Day 1

Day 0

*Single Cells*

Day 0

**A**

**rxn10 rnx11 rxn12 rxn13 rxn14 rxn15 rxn16 rxn17 rxn18**

**
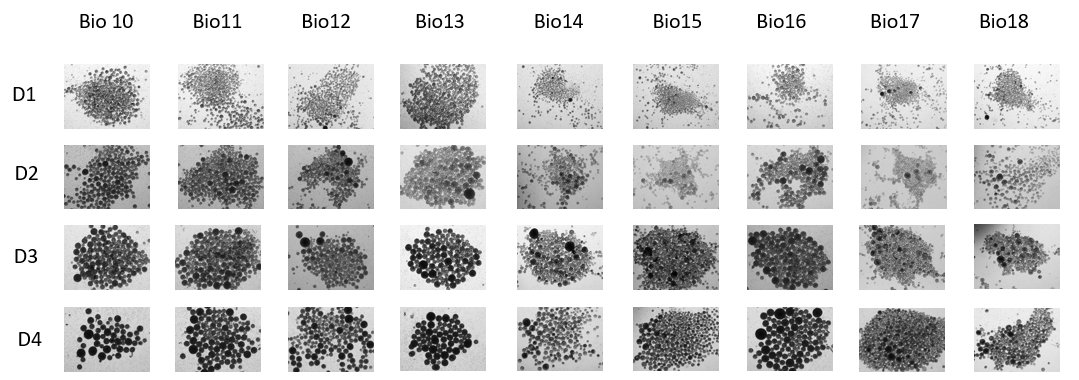
**

Batch 2

**iPSC Bioreactor Expansion**

**Cell Dissociation Time Study**

**B**

**D**

**
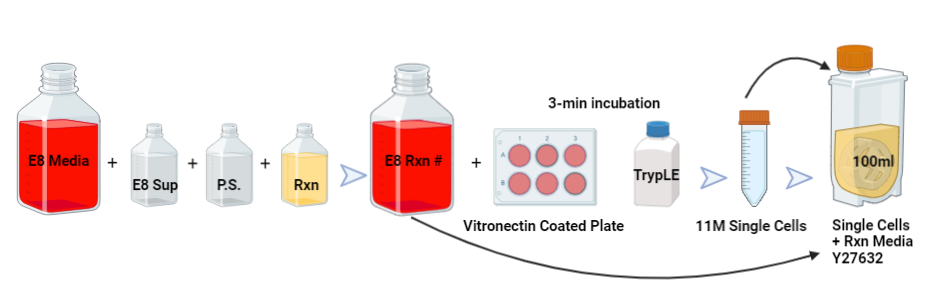
**

**Figure S2: Aggregate Outputs and Optimization of Bioreactor Process Parameters**

1. Daily aggregate images of all samples
2. Continuous growth rate curve after day 4 passaging between bioreactors
3. Digestion time optimization for optimal recovery rate between passages
4. Growth time optimization shows that passaging needs to be done on day 3-4 of culture.
5. A schematic of the method used to seed the bioreactors.

**
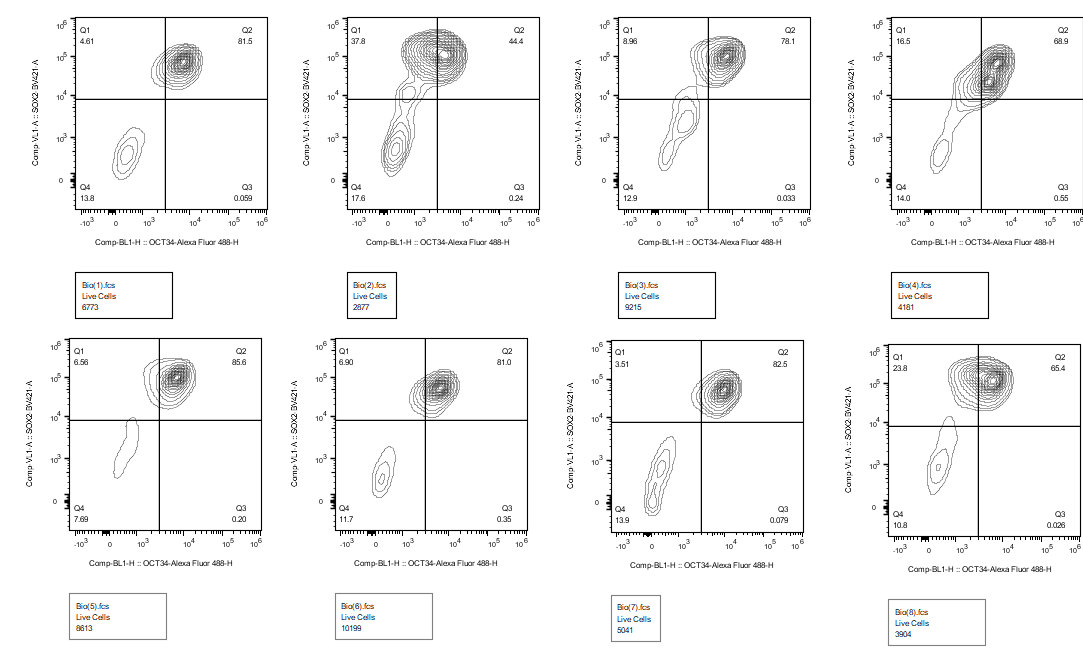

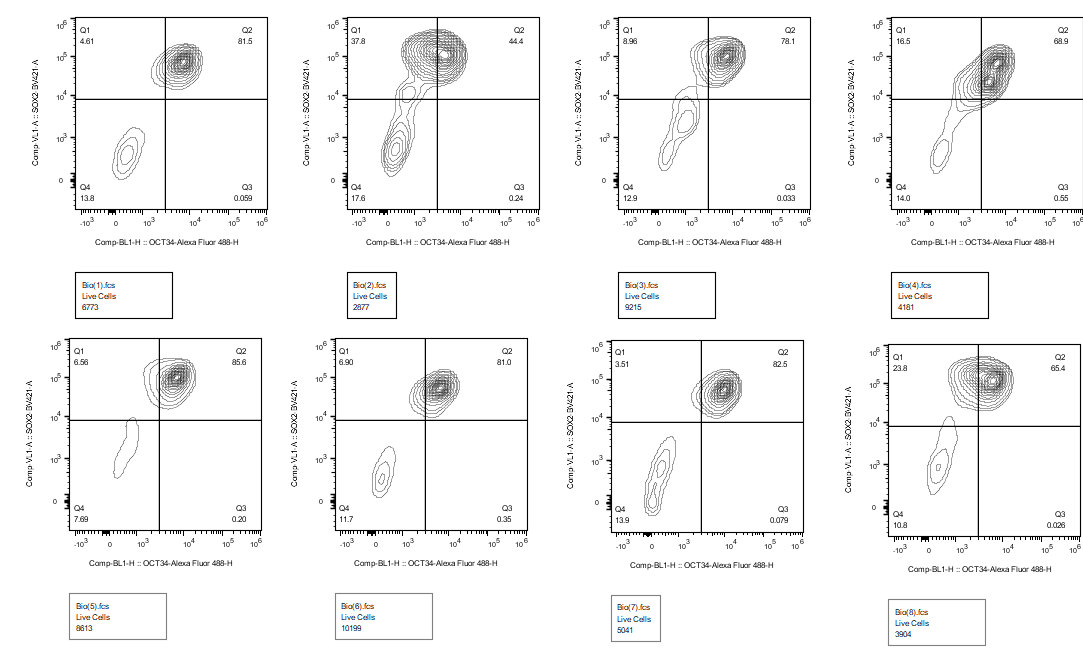

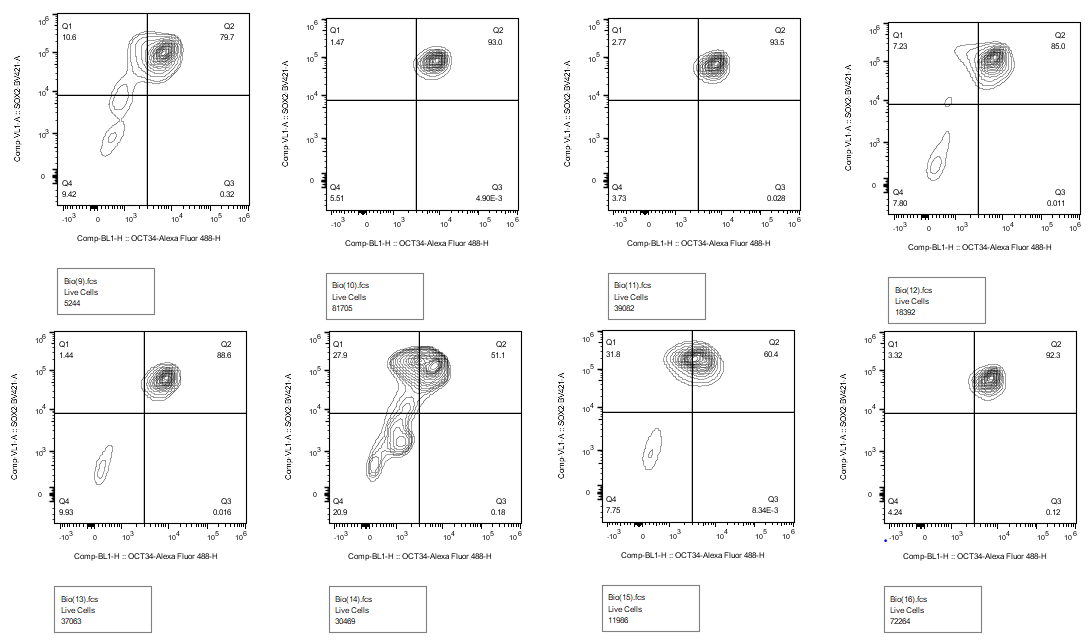

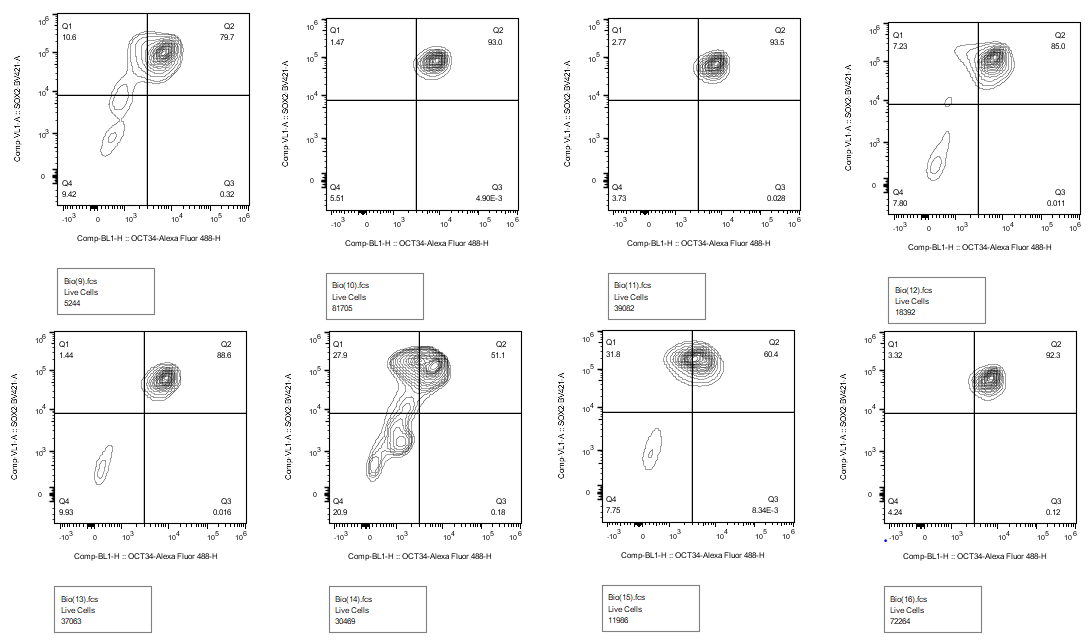

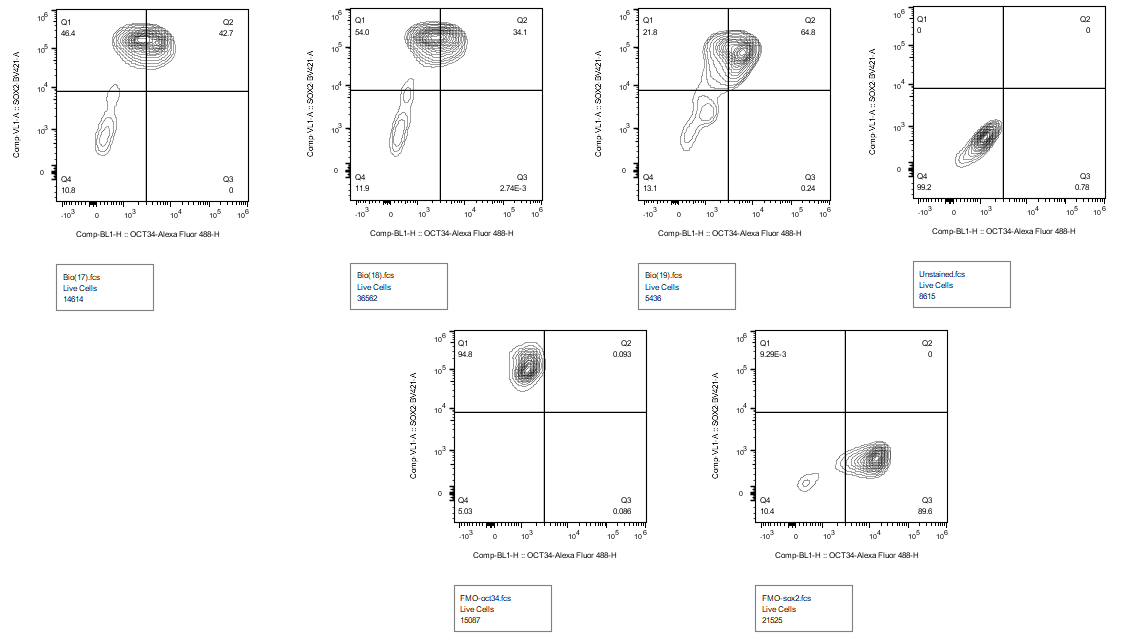
**

**A**

rxn1 rxn2 rxn3 rxn4

rxn5 rxn6 rxn7 rxn8

rxn9 rxn10 rxn11 rxn12

rxn13 rxn14 rxn15 rxn16

rxn17 rxn18 rxn19 Unstained

**
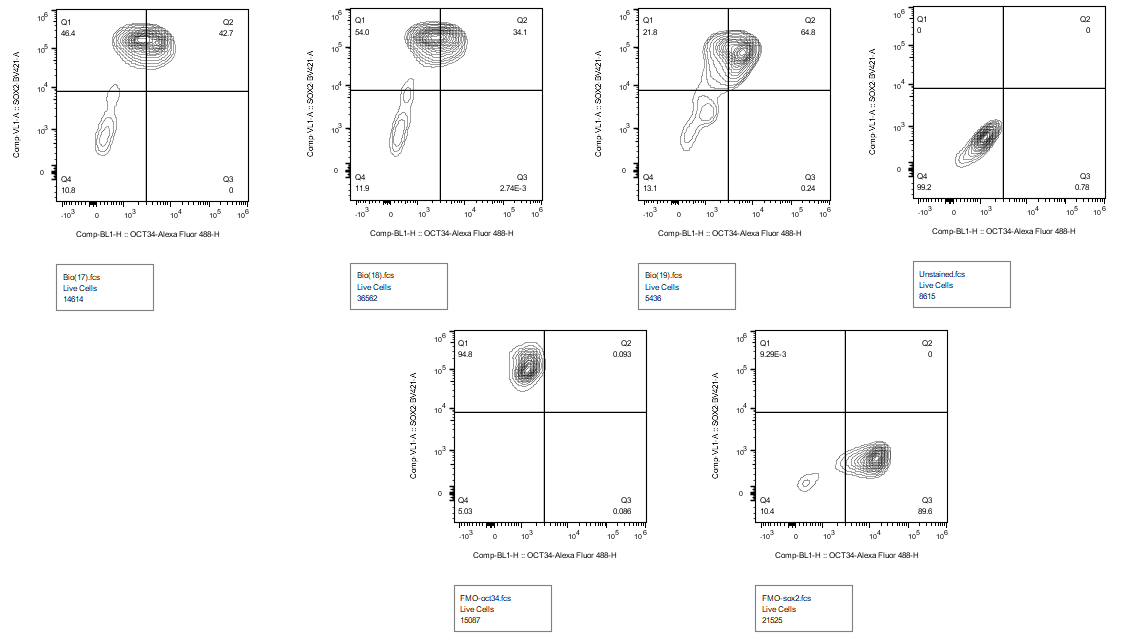
**

**Figure S3: Flow Intracellular Staining**

1. OCT4 and SOX2 Coexpression results

of all design reactions.

FMO OCT4 FMO SOX2

**
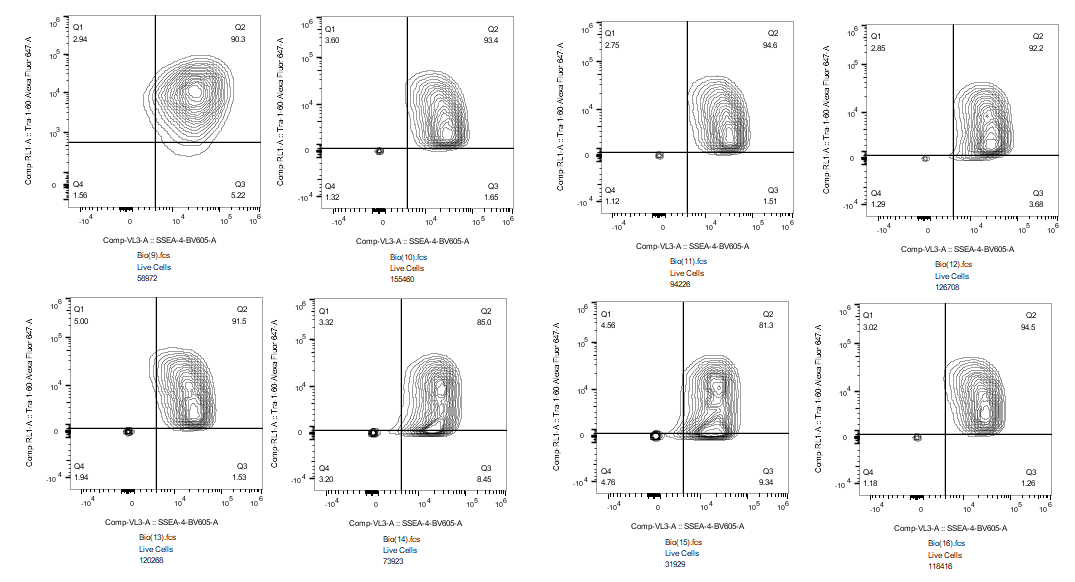

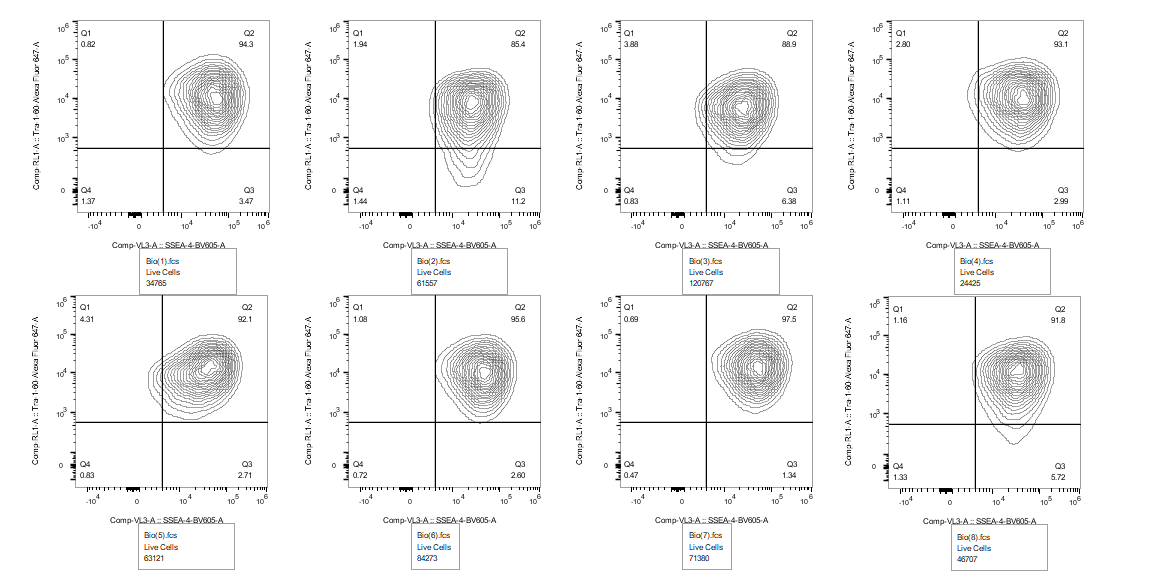

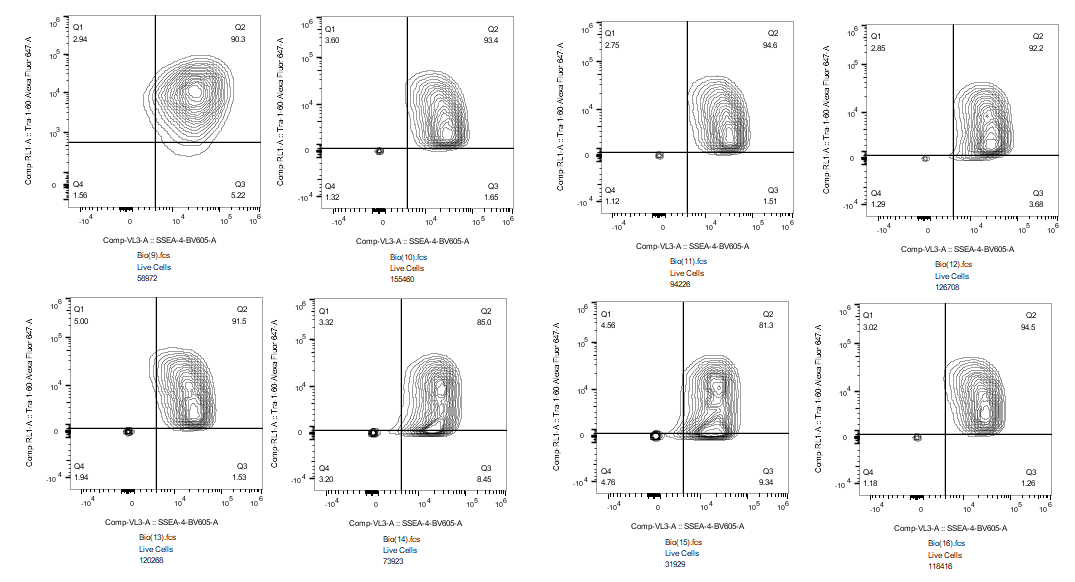

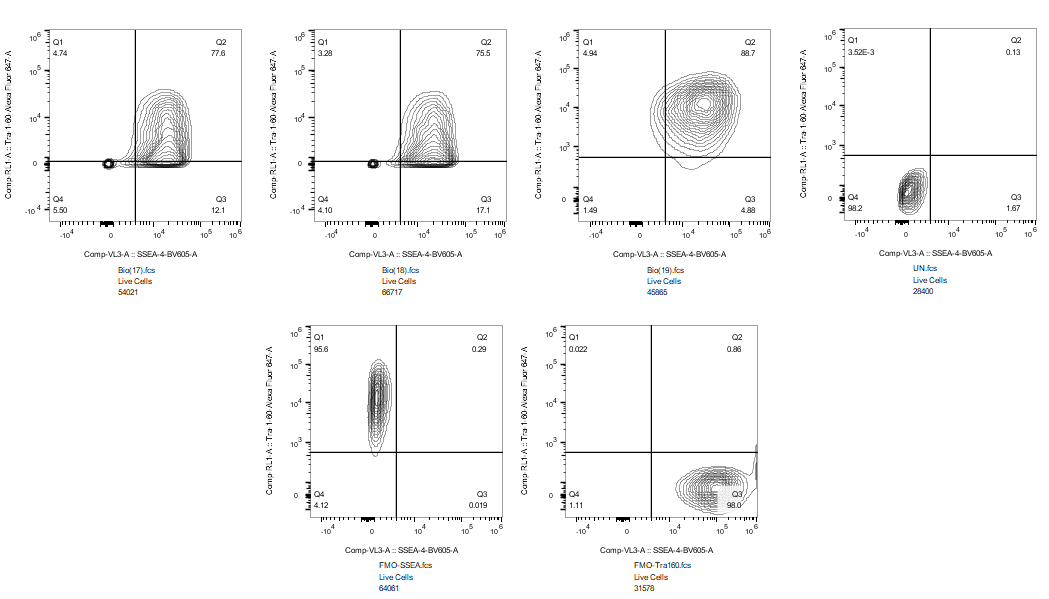

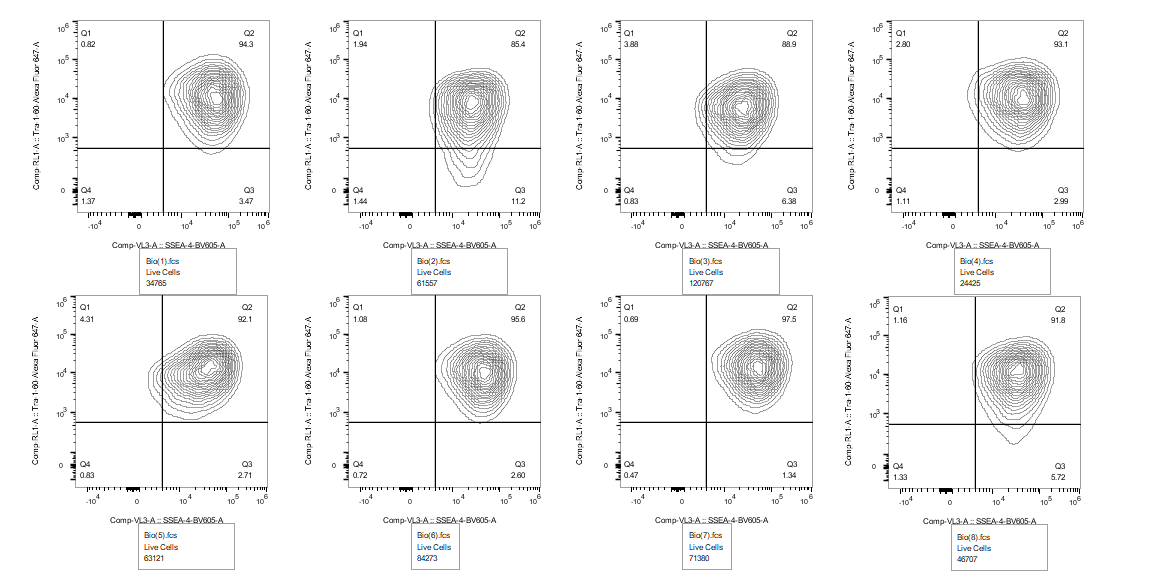
**

**A**

rxn1 rxn2 rxn3 rxn4

rxn5 rxn6 rxn7 rxn8

rxn9 rxn10 rxn11 rxn12

rxn13 rxn14 rxn15 rxn16

rxn17 rxn18 rxn19 Unstained

**
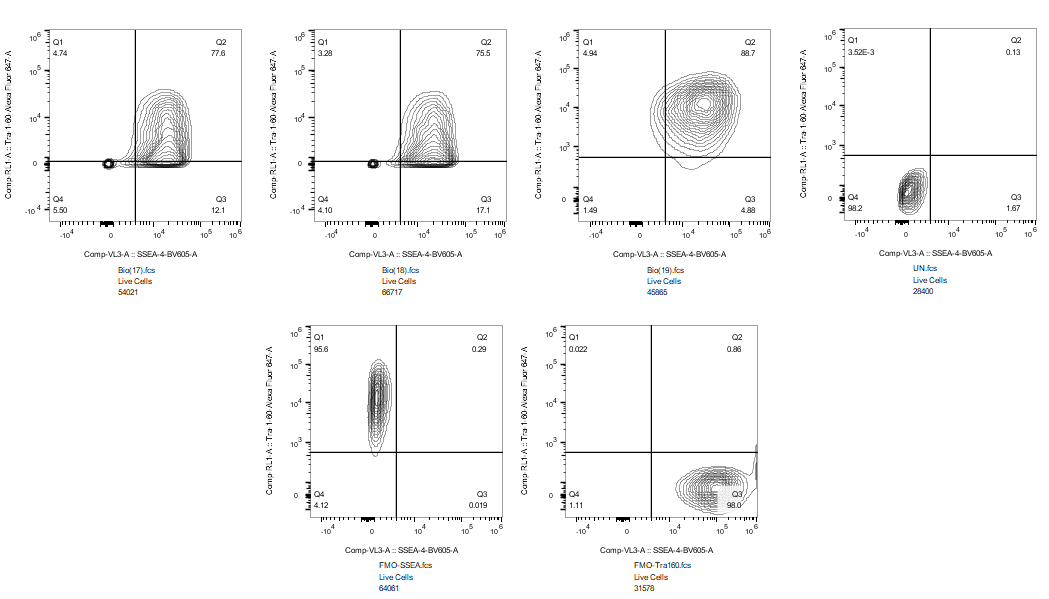
**

**Figure S4: Flow Extracellular Staining**

1. SSEA4 and TRA-1-60 Coexpression

results of all design reactions.

FMO SSEA4 FMO TRA-1-60


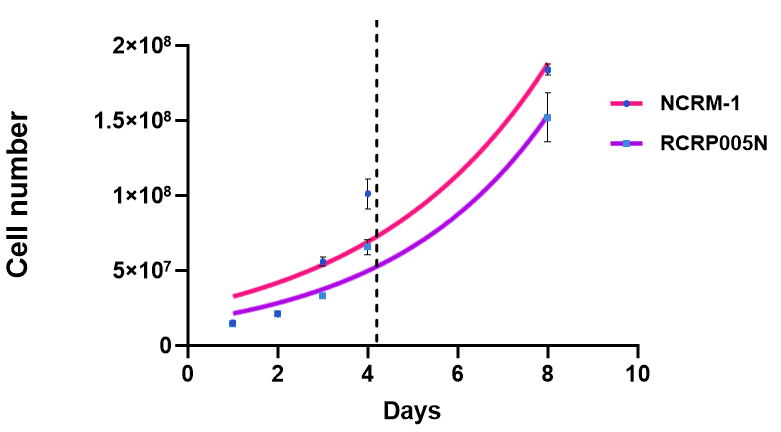

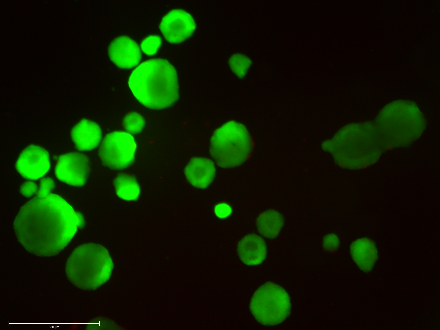

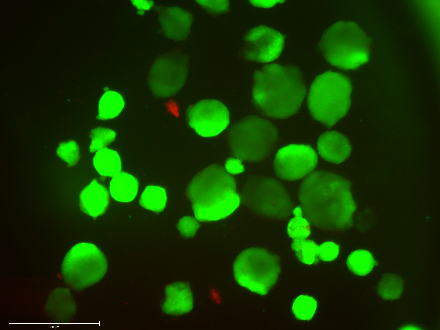

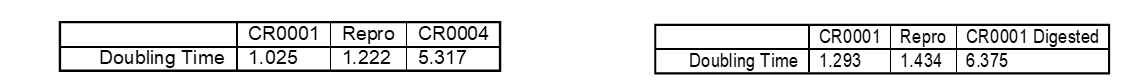

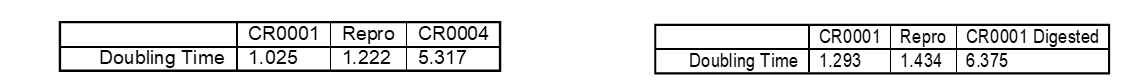

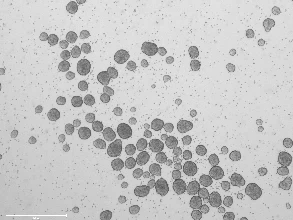

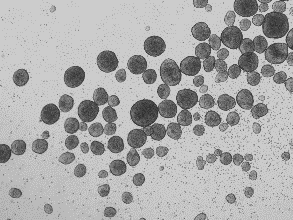

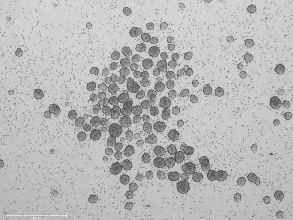

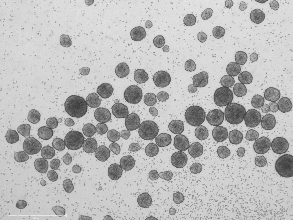

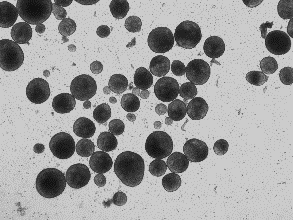

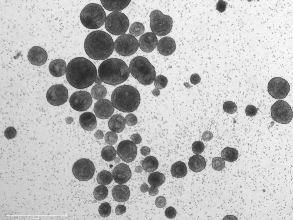

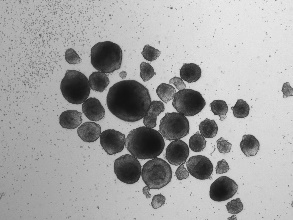

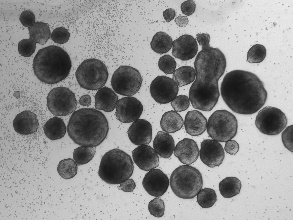

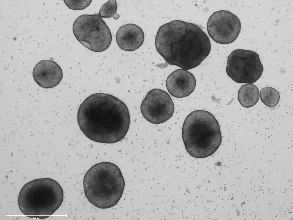

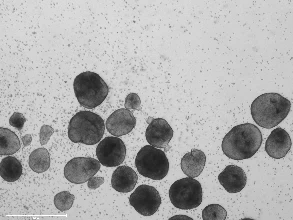

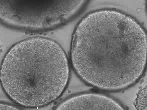

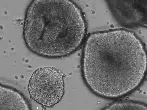


20x

20x

**650µm**

**RCRP005N**

**NCRM-1**

NCRM-1

RCRP005N

NCRM-1

RCRP005N

**FDA/PI**

**FDA/PI**

**SOX2**

**97.8%**

**OCT4**

**91.8%**

**OCT4**

**97.4%**

**SOX2**

**98.4%**

**D**

**C**

**A**

D1

D2

D4

D5

D8

**B**

Passage


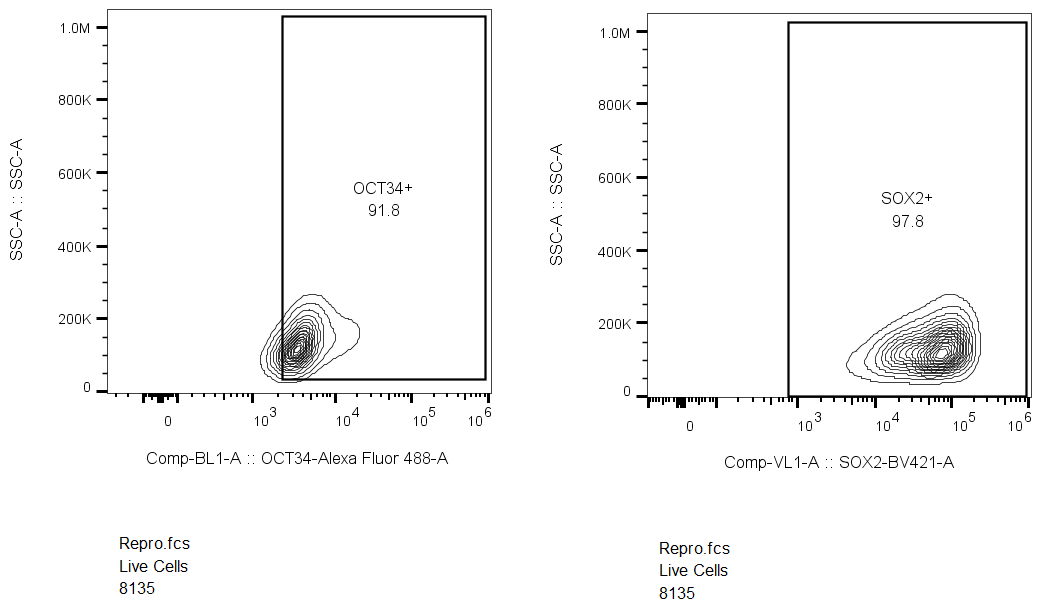


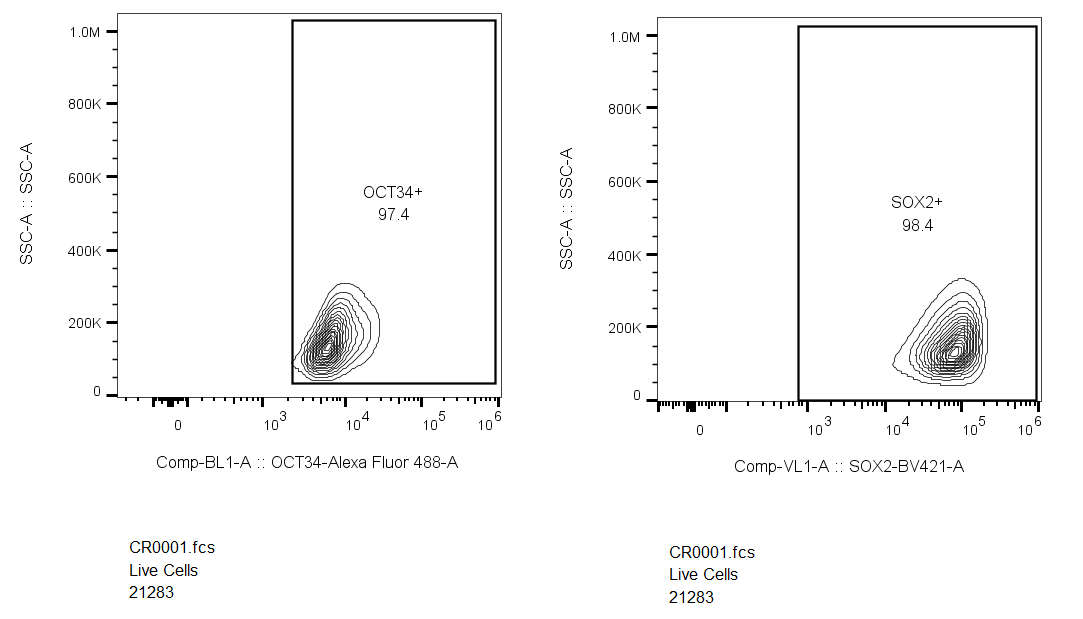


**Figure S5: Validation with different cell line and passaging**

1. Aggregate images throughout the culture assay on two cell lines
2. Flow Cytometry data on OCT4 and SOX2 intracellular markers on day 8 of culture
3. Measured growth curve before and after passaging
4. Live/Dead staining on day 8 of culture using Fluorescein diacetate (FDA) for live cells and Propidium iodide (PI) for dead cells.

**A**


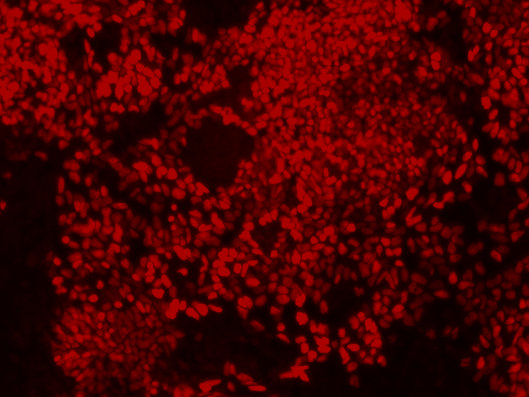

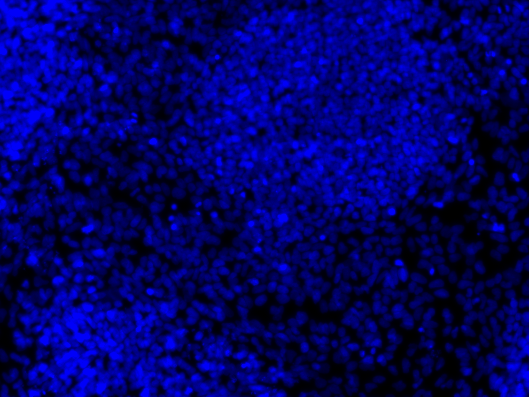

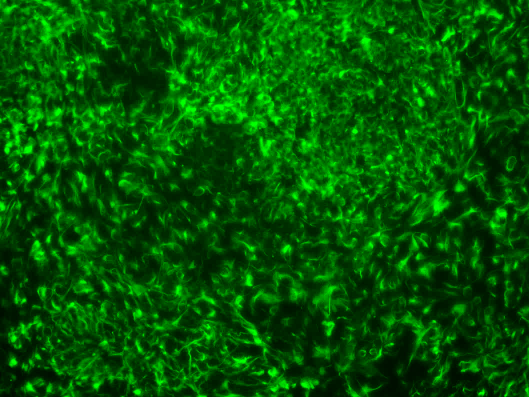


**PAX6**

**Nestin**

**DAPI**

**200µm**


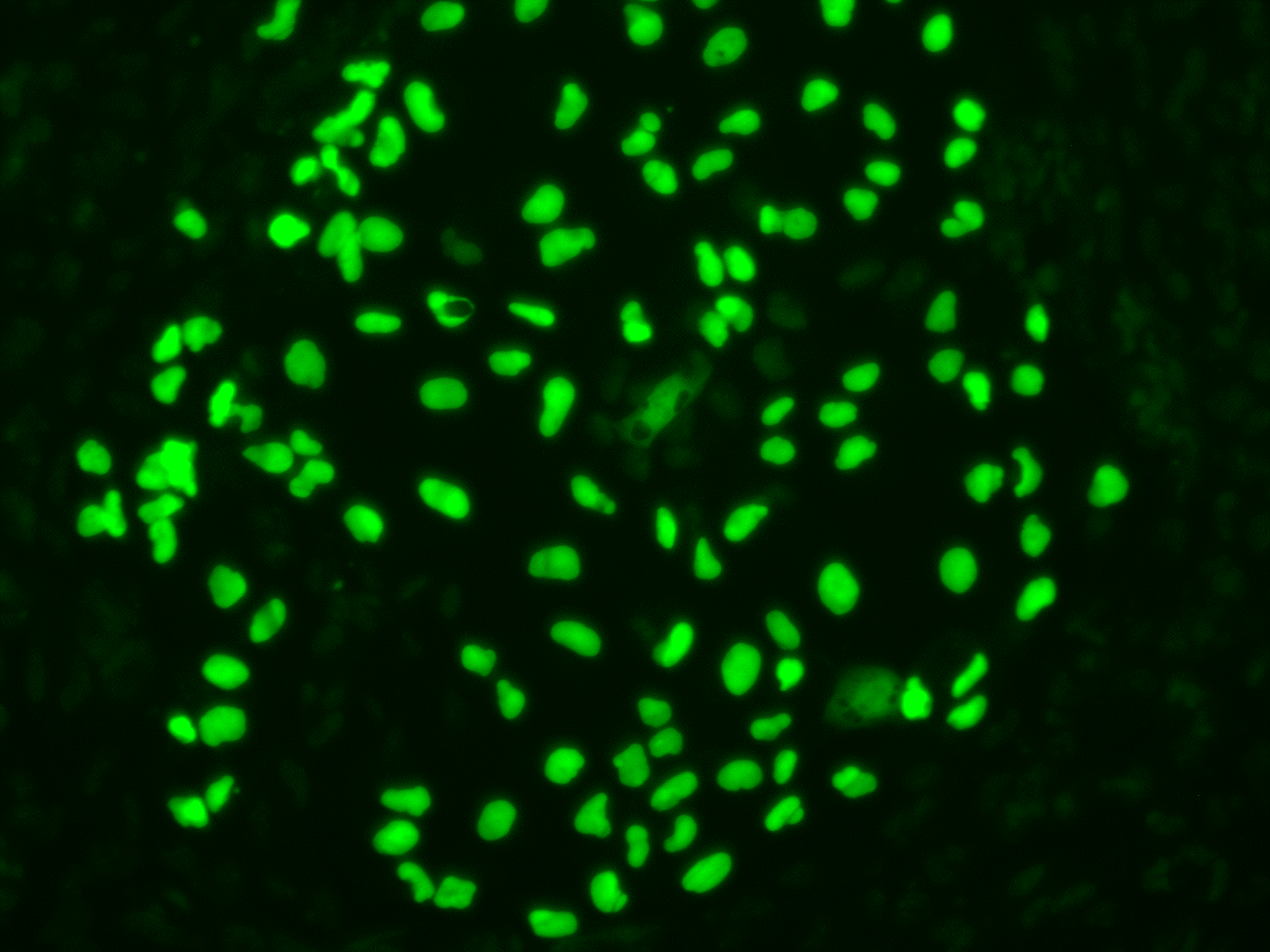

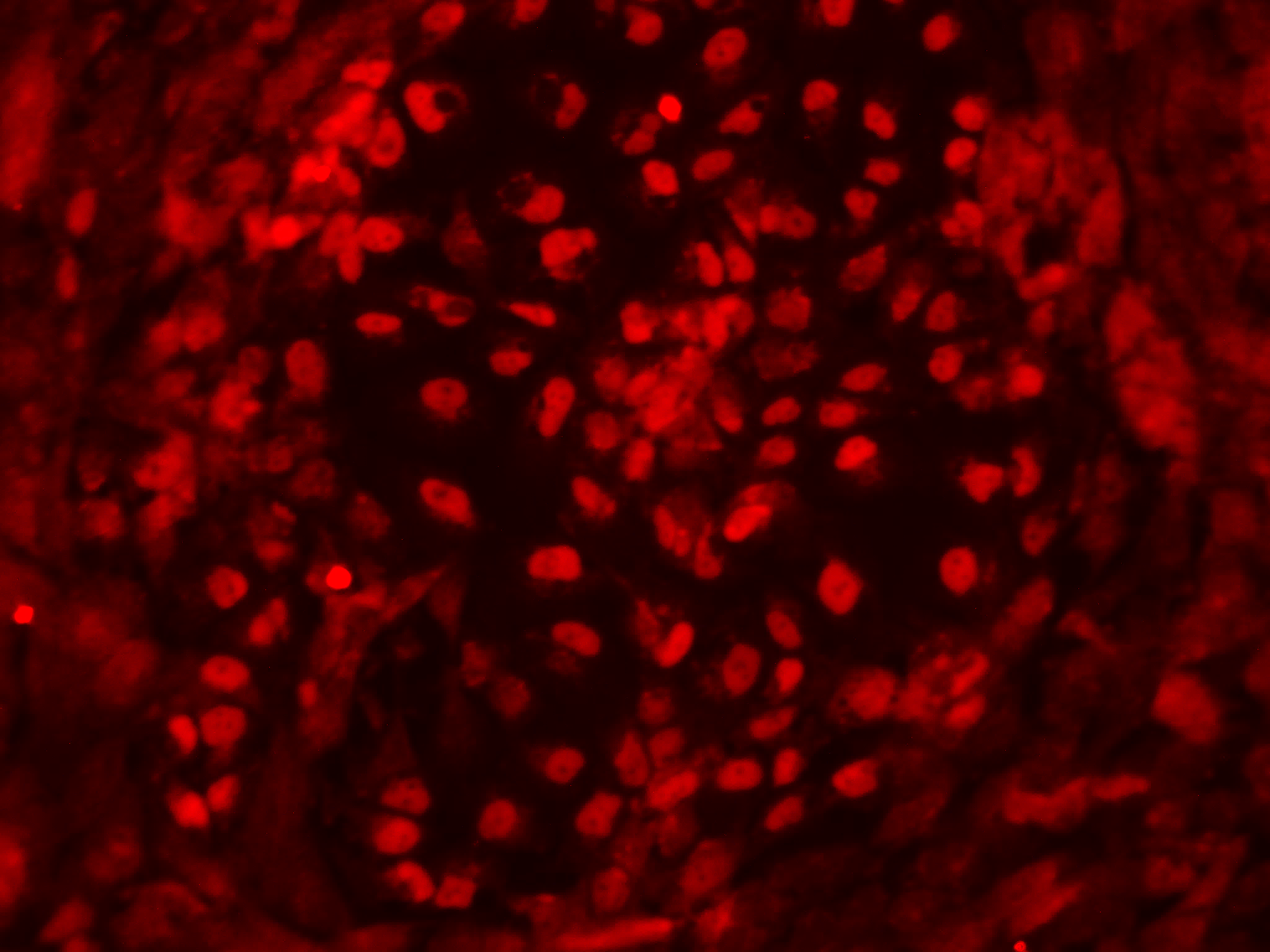

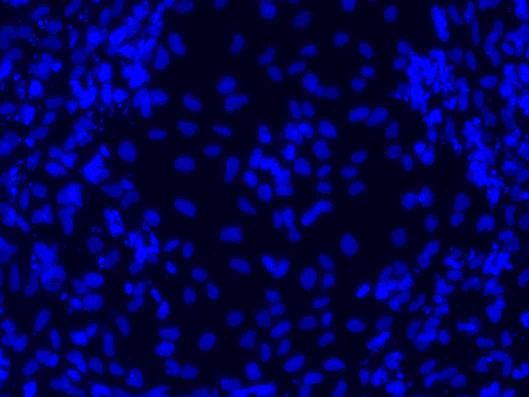

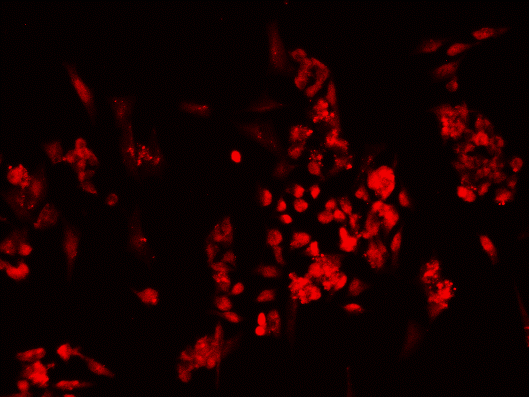

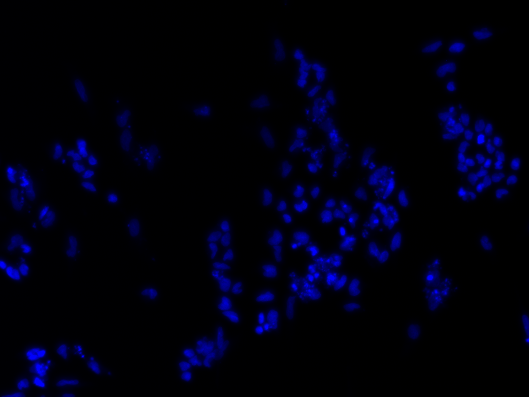


**SOX17**

**FOXA2**

**DAPI**

**B**

**Brachyury (T)**

**DAPI**


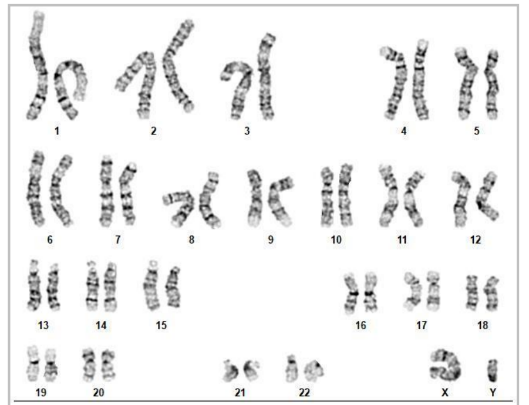


**Figure S6: Tri-lineage differentiation and Karyotyping stability**

1. Tri-lineage differentiation validation on the passaged cells

*Note: For mesoderm the same cells were stained for TNNT2 and (T) but on different wells since they both had the same primary antibody host

1. Karyotyping results of the cells that were cultured in the optimized medium.

**DAPI**


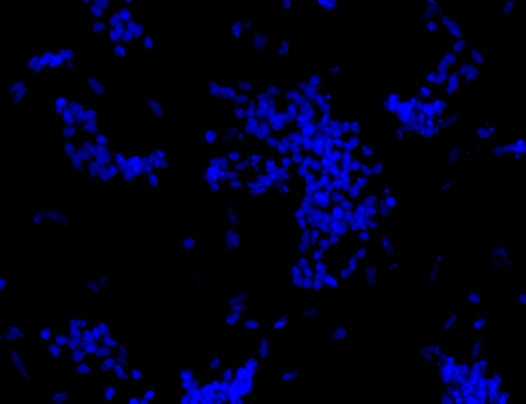

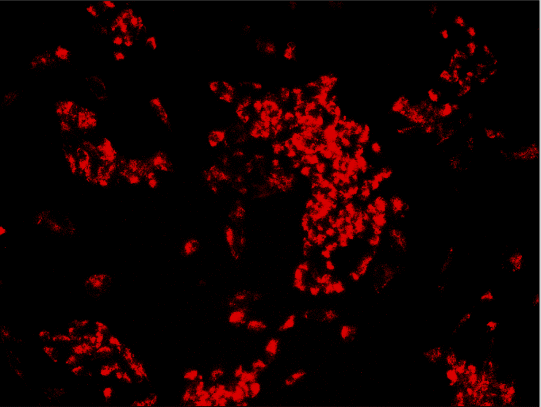


**TNNT2**

**
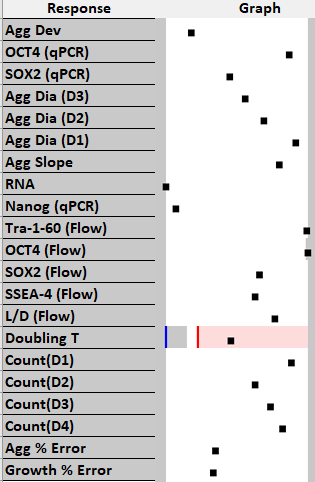

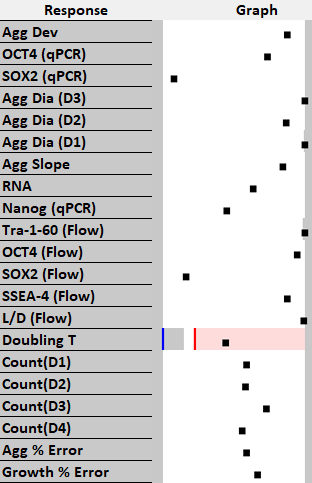

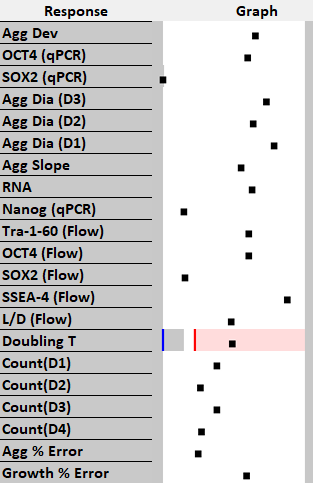

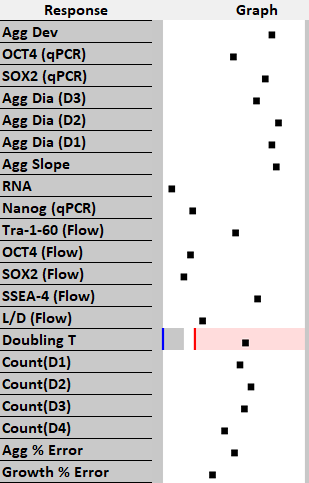

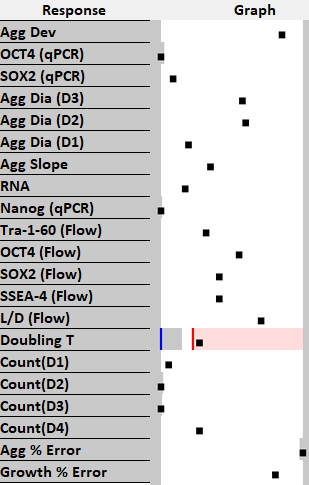
**

**A**

**PEG Set Point**

**PVA Set Point**

**Heparin Set Point**

**Pluronic F68 Set Point**

**Dextran Sulfate Set Point**

**Figure S6: Individual Component Set Point Analysis**

1. Component set point analysis using MODDE showing individual effector impact on the responses measured.

**
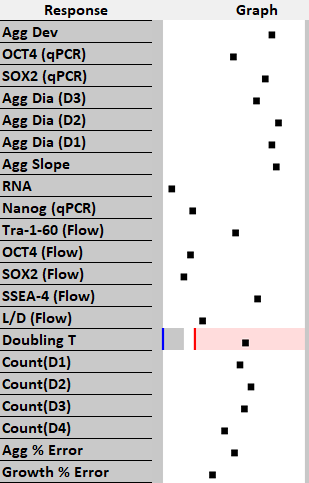

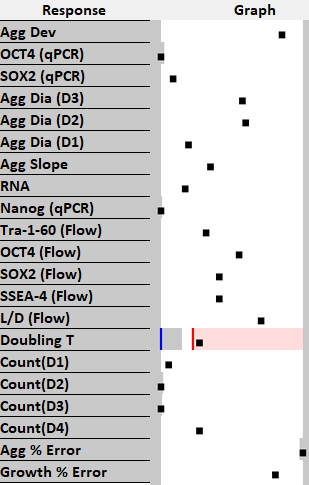
**

**Pluronic F68 Set Point**

**Dextran Sulfate Set Point**

**Figure S7: Individual Component Set Point Analysis**

1. Component set point analysis using MODDE showing individual effector impact on the responses measured.

**Supplemental Tables:**

**Table S1: List of Primers used for qPCR.**

| **Gene** | **Forward Primer** | **Reverse Primer** |
| --- | --- | --- |
| Nanog | GGATGGTCTCGATCTCCTGA | CCTCCCAATCCCAAACAATA |
| SOX2 | GCTACAGCATGATGCAGGACCA | TCTGCGAGCTGGTCATGGAGTT |
| POU5F1 | GAACCAGTATCGAGAACCG | TCAGTTTGAATGCATGGGAG |
| GAPDH | TATCGTGGAAGGACTCATGACC | TAGAGGCAGGGATGATGTTCTG |

**Table S2: List of antibodies and reagents used.**

| **ANTIBODIES** | **SOURCE** | **IDENTIFIER** |
| --- | --- | --- |
| POU5F1 | Santa Cruz Biotechnology | Cat# sc-5279: RRID:AB_628051 |
| SSEA4 | DSHB | Cat#MC-813-70: RRID:AB_528477 |
| SOX2 | Abcam | Cat# ab5603: RRID:AB_304980 |
| NANOG | Thermo Fisher Scientific | Cat# PA1-097: RRID:AB_2539867 |
| 488-Donkey-a-Mouse | Jackson Immuno Research | Cat# 715-546-151, RRID:AB_2340850 |
| 594-Donkey-a-Rabbit | Jackson Immuno Research | Cat# 711-586-152, RRID:AB_2340622 |
| Alexa Fluor® 488 Mouse anti-Oct3/4 | BD Biosciences | Cat# 560253, RRID:AB_1645304 |
| BV605 Mouse Anti-SSEA-4 | BD Biosciences | Cat# 563119, RRID:AB_2738015 |
| Brilliant Violet 421™ anti-SOX2 Antibody | Biolegend | Cat# 656114, RRID:AB_2566262 |
| Alexa Fluor® 647 Mouse anti-Human TRA-1-60 Antigen | BD Biosciences | Cat# 560850, RRID:AB_10565983 |
| **REAGENTS** | | |
| Essential 8™ Flex | ThermoFisher Scientific | A2858501 |
| Vitronectin | Thermo Fisher Scientific | A31804 |
| Y-27632 dihydrochloride | MedChem Express | HY-10583 |
| DPBS (without calcium and magnesium) | Thermo Fisher Scientific | 14190144 |
| TrypLE | Thermo Fisher Scientific | 12563-029 |
| Accutase | Thermo Fisher Scientific | A1110501 |
| **CELL LINES** | | |
| RCRP5005N* | REPROCELL | StemRNA™ Human iPSC 771-3G |
| NCRM-1** | iXCells | CR0000001 |

* Established from endothelial progenitor cells from peripheral blood. Reprogrammed with the Stemgent StemRNA 3rd Gen Reprogramming technology.

** Established from CD34+ cord blood cells. Reprogrammed with Episomal plasmid method.
